# A Predictive Mathematical Modeling Approach for the Study of Doxorubicin Treatment in Triple Negative Breast Cancer

**DOI:** 10.1038/s41598-017-05902-z

**Published:** 2017-07-18

**Authors:** Matthew T. McKenna, Jared A. Weis, Stephanie L. Barnes, Darren R. Tyson, Michael I. Miga, Vito Quaranta, Thomas E. Yankeelov

**Affiliations:** 1 Vanderbilt University Institute of Imaging Science, Nashville, USA; 2 Department of Biomedical Engineering, Vanderbilt University, Nashville, USA; 3 Department of Biomedical Engineering, The University of Texas at Austin, Austin, USA; 4 Department of Cancer Biology, Vanderbilt University School of Medicine, Nashville, USA; 5 Department of Radiology & Radiological Sciences, Vanderbilt University School of Medicine, Nashville, USA; 6 Department of Diagnostic Medicine, Dell Medical School, The University of Texas at Austin, Austin, USA; 7 Institute for Computational and Engineering Sciences, The University of Texas at Austin, Austin, USA; 8 Livestrong Cancer Institutes, The University of Texas at Austin, Austin, USA

## Abstract

Doxorubicin forms the basis of chemotherapy regimens for several malignancies, including triple negative breast cancer (TNBC). Here, we present a coupled experimental/modeling approach to establish an *in vitro* pharmacokinetic/pharmacodynamic model to describe how the concentration and duration of doxorubicin therapy shape subsequent cell population dynamics. This work features a series of longitudinal fluorescence microscopy experiments that characterize (1) doxorubicin uptake dynamics in a panel of TNBC cell lines, and (2) cell population response to doxorubicin over 30 days. We propose a treatment response model, fully parameterized with experimental imaging data, to describe doxorubicin uptake and predict subsequent population dynamics. We found that a three compartment model can describe doxorubicin pharmacokinetics, and pharmacokinetic parameters vary significantly among the cell lines investigated. The proposed model effectively captures population dynamics and translates well to a predictive framework. In a representative cell line (SUM-149PT) treated for 12 hours with doxorubicin, the mean percent errors of the best-fit and predicted models were 14% (±10%) and 16% (±12%), which are notable considering these statistics represent errors over 30 days following treatment. More generally, this work provides both a template for studies quantitatively investigating treatment response and a scalable approach toward predictions of tumor response *in vivo*.

## Introduction

When cytotoxic therapy was first applied to cancer, few principles existed to guide its use^1^. Skipper provided a framework through the formulation of the log-kill hypothesis, postulating that a given dose of chemotherapy would kill a fixed fraction of tumor cells regardless of tumor size^2^. Based on this framework, a systemic chemotherapy paradigm was established, in which cytotoxic agents were administered several times, even after disease could no longer be detected. Following this, investigators sought to improve response through dose escalation, but their efforts were met with limited improvement in tumor response^3, 4^. Dosing paradigms were updated after Norton and colleagues hypothesized that tumor kill is proportional to tumor growth rate^5^. This led to development of dose-dense schedules, which decrease the time between doses to target smaller, faster-growing tumors. These dose-dense schedules resulted in a significant improvement over previous treatment protocols^6^ and remain the standard-of-care for triple negative breast cancer (TNBC) treatment. In recent years, several theoretical models have been developed to further refine treatment regimens^7^. Of note, Gatenby and colleagues proposed an adaptive model which adjusts doses based on tumor volume changes^8, 9^. Metronomic dosing schedules advocate smaller, more frequent dosing^10, 11^. These new dosing approaches are predicated on both the timing of therapy administration and response evaluation^12^ but have revealed a fundamental limitation in the current understanding of the pharmacokinetic (PK) and pharmacodynamic (PD) properties of cytotoxic agents. While the potency, efficacy, and mechanism of action of these agents have been the target of study for years, these pharmacologic properties are inherently insufficient to predict the spatiotemporal response of individual tumors to treatment, limiting the ability to realize these theoretical dosing schedules.

In this contribution, we propose a scalable experimental/modeling framework that incorporates the dynamics of therapy and response. In this way, we hope to complement theoretical dosing models with a precise approach to scale *in vitro* observations to *in vivo* experiments. The utility of this framework is demonstrated in the context of doxorubicin treatment in TNBC. Doxorubicin is a standard-of-care, DNA-damaging agent used in the treatment of a host of malignancies, including TNBC^13–15^. As we review below, the current approaches to the study of doxorubicin are insufficient to generate temporally-resolved predictions of TNBC response to time-varying doxorubicin treatments.

Cellular response to a given therapeutic is often evaluated by one of a variety of *in vitro* assays and generally interpreted using dose-response curves. In these assays, drug is typically applied to a cell population over a wide range of concentrations. Following a predefined treatment time (usually 72 hours) drug effect is quantified with one of many end-point assays that measure the number of viable cells (often indirectly). These data are then analyzed with the Hill equation, a sigmoidal function that is used to describe the relationship between drug concentration and drug effect^16^. The Hill equation contains a number of free parameters including: the maximal drug effect (*E*_*max*_), the concentration of drug that yields a half-maximal effect (*EC*_*50*_), the effect in the absence of drug (*E*_*0*_), and the Hill coefficient (*h*), which describes the slope of the dose response curve. The parameters that result from the best fit of the model to the dose-response curves are specific to each cell line, and those data are used to guide drug dosing for subsequent *in vivo* experiments. While this approach has great merit in evaluating drug efficacy and identifying new therapeutics, it necessarily overlooks the importance of the relative timing of treatments and response measurement. Further, slight changes in experimental duration or growth conditions have been shown to significantly impact estimation of model parameters^17, 18^. Even proposed metrics that analyze population rates of change to correct for varying cell line behaviors and experimental protocols assume a constant population rate of change following application of therapy^17, 18^. Consequently, the predictive potential of such approaches is fundamentally limited, particularly in the setting of cytotoxic agent use *in vivo*, in which agents are applied as impulses and resilient populations, which demonstrate temporally-varying population growth rates following therapy, are often observed.

Relative to the efficacy studies above, the temporal relationship between cytotoxic treatment and its effects has received little attention. Eichholtz-Wirth and colleagues first demonstrated the dependence of cell survival on doxorubicin exposure time, deriving an empirical relationship between surviving fraction of cells (*SF*), drug concentration (*c*), and length of exposure (*t*), through a sensitivity constant (*k*): *SF* = *e* ^−*ktc*^ ^19^. Others have proposed modifications to the classic Hill function to incorporate drug exposure times^20, 21^. To resolve the temporal dynamics of the cellular response to therapy, Lobo and Balthazar proposed a transit compartment model to describe the relationship between drug application and the time lag until drug effects were realized^22^. These models were all built utilizing end-point assays evaluating the percent survival following various exposure times. Lankelma employed a host of clonogenic assays following treatment with various concentrations of doxorubicin for multiple exposure times^23, 24^. They quantified cell population size over time and constructed a model relating treatment parameters to these cell population dynamics. However, a model of therapy response that incorporates both the dynamics of therapy (pharmacokinetics) as well as the dynamics of cellular response (pharmacodynamics) has remained elusive. Such modeling would represent a critical advance, as it would allow more precise measurements of response and customization of treatment protocols following estimation of PK parameters.

**Figure 1:**
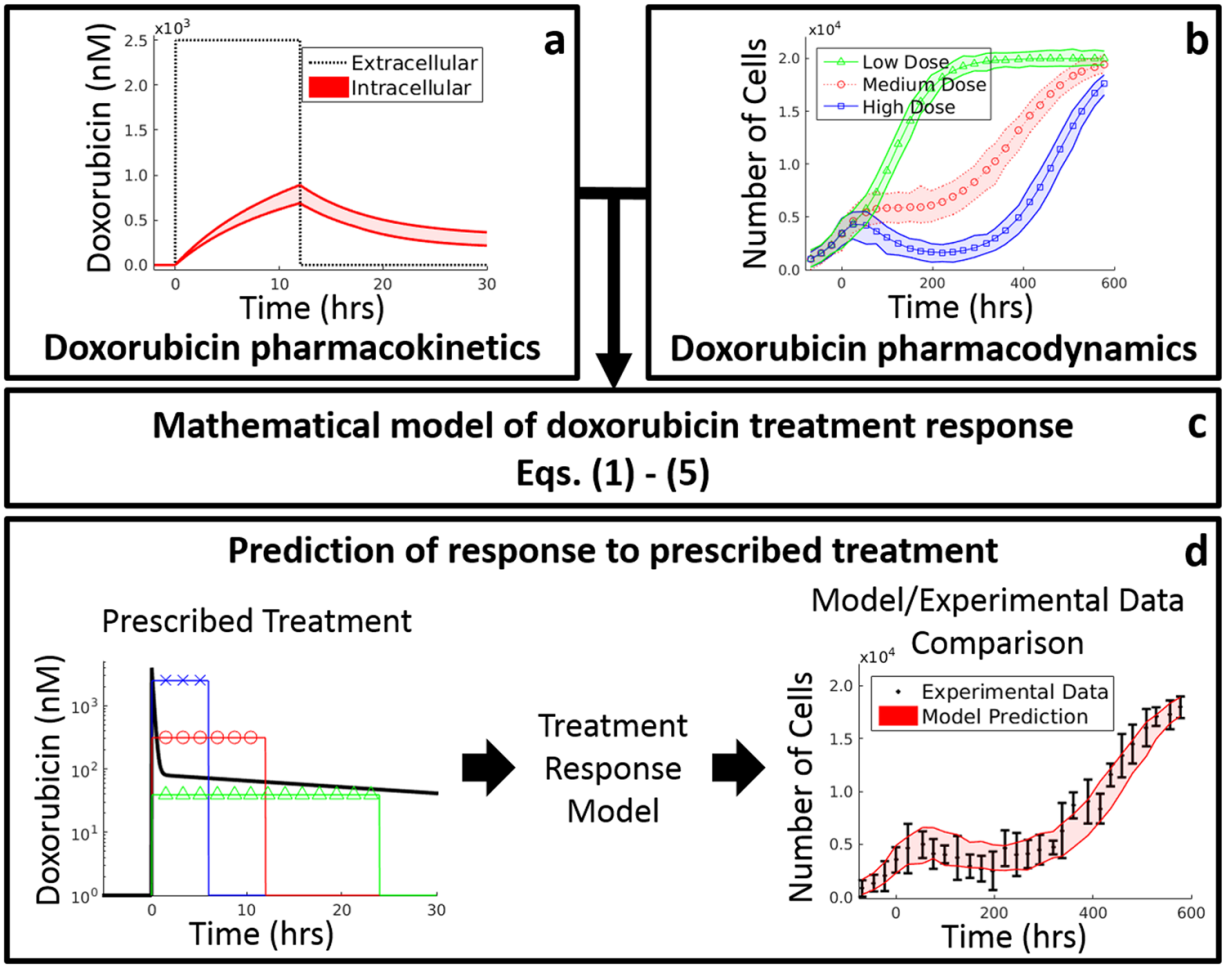
Overview of cell-line specific modeling framework for doxorubicin treatment response prediction. A series of time-resolved fluorescence microscopy experiments were performed to quantify both the uptake of doxorubicin into TNBC cell lines (**a**) as well as the response of those cell lines to various doxorubicin treatments (**b**). Data from these experiments were used to fit the model (i.e., Eqs (1–5)) of treatment response in TNBC (**c**). After training the model on observed data, the model can be initialized with a cell count and a prescribed treatment timecourse to predict cell population dynamics following the proposed treatment (**d**). These predictions can then be compared to experimental results.

This work focuses on the construction of a mathematical model to predict TNBC cell population dynamics in response to time-varying doxorubicin treatments. The approach outlined below incorporates a series of experiments in a panel of four TNBC cell lines designed to measure both the *in vitro* pharmacokinetics (PK) and pharmacodynamics (PD) of doxorubicin therapy. The PK/PD parameters are quantified through time-resolved fluorescent microscopy, and these data are used to drive the development of a treatment response model. This approach yields a mathematical model of doxorubicin therapy with distinct parameter value sets for each TNBC cell line. This model can generate hypotheses that are directly testable in both the *in vitro* and *in vivo* settings. Thus, the objectives of this contribution are to: (1) establish a model that describes *in vitro* doxorubicin pharmacokinetics, (2) establish a model relating treatment variables (concentration and duration) to subsequent cell population dynamics, and (3) propose a prediction scheme leveraging doxorubicin pharmacokinetic and pharmacodynamic data to predict response to various doxorubicin treatments (Fig. 1).

## Methods

### Cell culture

TNBC is a subgroup of invasive cancers that lack significant expression of the estrogen receptor, progesterone receptor, and human epidermal growth factor receptor 2^25^. Lacking specific receptor targets, the current approach to adjuvant and neoadjuvant therapy (NAT) for locally advanced TNBC utilizes a combination of cytotoxic drugs with a particular emphasis on doxorubicin, cyclophosphamide, and docetaxel^13–15^. Lehmann and colleagues identified six subtypes of TNBC: two basal-like subtypes, an immunomodulatory subtype, a mesenchymal subtype, a mesenchymal stem cell-like subtype, and a luminal subtype expressing androgen receptor^26, 27^. One cell line from four of these groups was selected for the current studies: MDA-MB-468 (basal-like 1), SUM-149PT (basal-like 2), MDA-MB-231 (mesenchymal), and MDA-MB-453 (luminal expressing androgen receptor). In selecting cell lines in this way, the proposed model of doxorubicin response can be assessed across a heterogeneous spectrum of TNBC cell lines.

All cell lines were obtained through American Type Culture Collection (ATCC, http://www.atcc.org) and maintained in culture according to ATCC recommendations. All cell lines were tested for mycoplasma after thawing using a PCR-based method (MycoAlert, Lonza, Allendale, NJ), and any positive cultures were immediately discarded. To facilitate automated image analysis for identifying and quantifying individual nuclei in the time-lapsed fluorescent microscopy experiments (described below), each of the four cell lines was modified to express a histone H2B conjugated to monomeric red fluorescent protein (HαmRFP; Addgene Plasmid 18982) as previously described^17, 28, 29^. Modified cells were grown in the same manner as their respective parental strains.

### Doxorubicin imaging and image processing

Time resolved fluorescent microscopy was employed to characterize the uptake of doxorubicin by each cell line. Doxorubicin is naturally fluorescent with excitation and emission peaks near 470 nm and 570 nm, respectively^30^. The intrinsic fluorescence of doxorubicin was leveraged to quantify the movement of doxorubicin from the extracellular space into cells. Each parental cell line was introduced into 96-well microtiter plates at ~10,000 cells per well. Each well was imaged at ~15 minute intervals *via* brightfield and fluorescent microscopy with a 20x objective in 2 × 2 image montages on a BD Pathway 855 Bioimager (BD Biosciences, San Jose, CA). Imaging began one hour prior to application of doxorubicin and continued for approximately 24 hours following doxorubicin application. An 8-fold range of doxorubicin concentrations, from 2500 nM to 312 nM, were applied to cells using a two-fold dilution series. After 6 or 12 hours, drug was removed *via* media replacement. Each of the ten conditions (i.e., four concentrations plus a control each at two exposure times) were collected in duplicate. These treatment conditions were designed to approximate drug exposure of human tumors *in vivo* as measured by the area under the doxorubicin concentration-time curve (a range of 1875 to 30000 nM∙hr was used experimentally to approximate the 4427 ± 418 nM∙hr observed *in vivo* ^31^) and peak doxorubicin concentration (312 to 2500 nM experimentally to approximate the 1000 to 5000 nM observed *in vivo* ^31^).

Digital images were segmented into extracellular and intracellular compartments through a hybrid, semi-automated process. Prior to doxorubicin application, segmentation was performed exclusively on the brightfield images to identify cell boundaries. Following application of doxorubicin, segmentation was performed on the fluorescent images with a threshold-based approach. See Supplementary Materials for details.

### Doxorubicin compartment modeling

**Figure 2:**
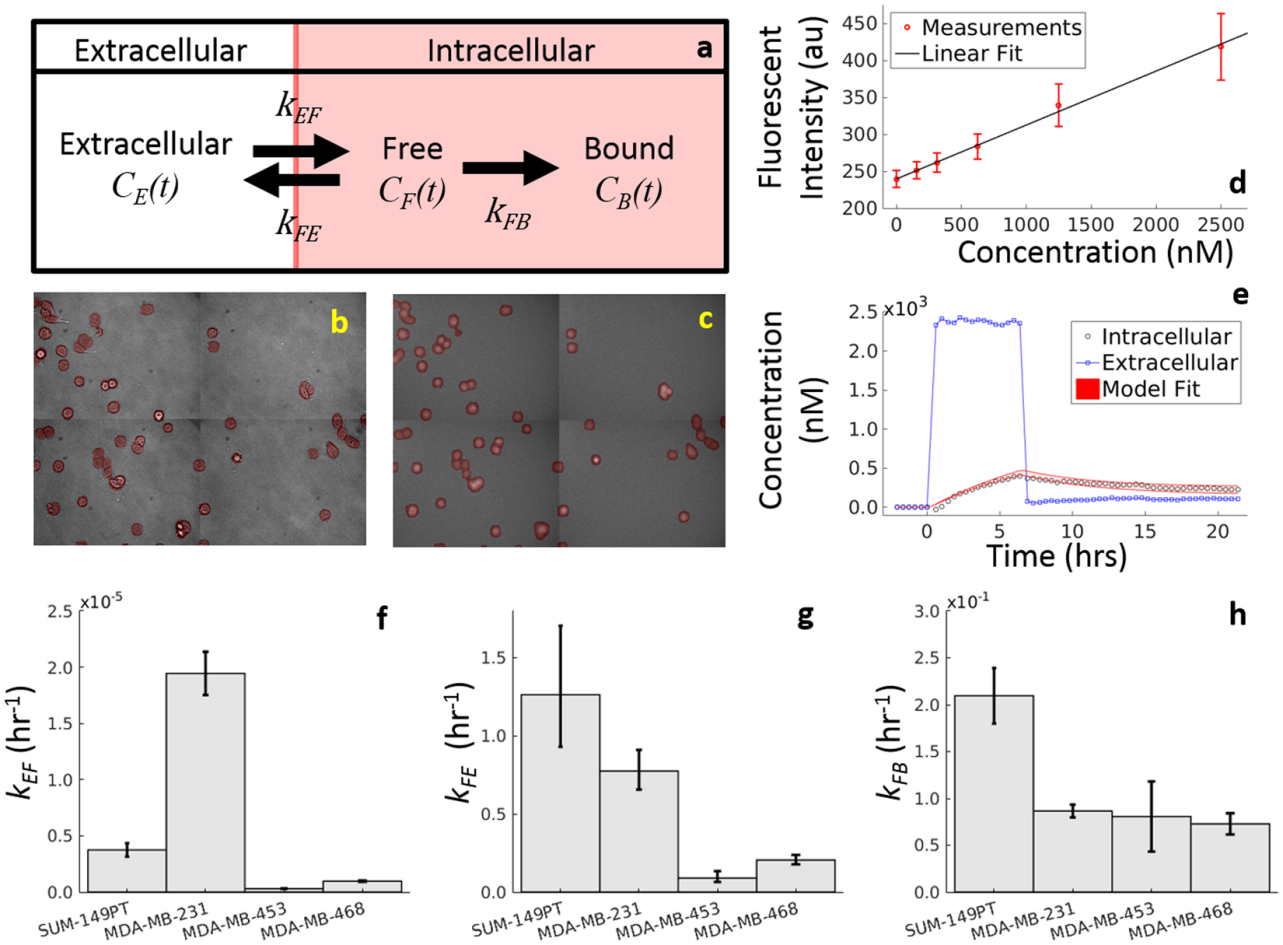
Overview of Doxorubicin Compartment Modeling. Doxorubicin pharmacokinetics is described with a three compartment model, illustrated in (**a**) and described by Eqs (1–3). To parameterize this model, each cell line is serially imaged *via* brightfield (**b**) and fluorescent microscopy (**c**) to monitor doxorubicin concentration over time. Images are separated into extracellular and intracellular (red overlay) compartments. As fluorescence intensity is proportional to doxorubicin concentration (**d**), the image intensities are converted into concentration, and extracellular and intracellular concentration timecourses are extracted from these images (**e**). Finally, the model is fit to these timecourses (**e**), and the model fit with 95% confidence interval are overlaid on the data. Experimentally-derived model parameter values with 95% CIs are reported for each TNBC cell line investigated (**f**–**h**).

A three compartment model was employed to describe the uptake and binding of doxorubicin in cancer cells. Briefly, doxorubicin is thought to enter cells *via* diffusion, possibly through a saturable carrier-mediated process^32, 33^. Once in the cell, doxorubicin is translocated to the nucleus where it intercalates DNA and stabilizes the topoisomerase II complex^34, 35^. Doxorubicin may also be actively effluxed from the cell *via* p-glycoprotein^36^. This process is modeled *via* mass conservation in Eqs (1–3):

1 $$\frac{d{C}_{E}(t)}{dt}={k}_{FE}\frac{{v}_{I}}{{v}_{E}}{C}_{F}(t)-{k}_{EF}{C}_{E}(t)$$

2 $$\frac{d{C}_{F}(t)}{dt}={k}_{EF}\frac{{v}_{E}}{{v}_{I}}{C}_{E}(t)-{k}_{FE}{C}_{F}(t)-{k}_{FB}{C}_{F}(t)$$

3 $$\frac{d{C}_{B}(t)}{dt}={k}_{FB}{C}_{F}(t)$$

where *C*_*E*_(*t*), *C*_*F*_(*t*), and *C*_*B*_(*t*) are the concentrations of doxorubicin in the extracellular, free, and bound compartments, respectively, at time *t*. Both the free and bound compartments were defined to share the same physical space (intracellular). The free compartment represents drug that has diffused into the cell, while the bound compartment represents drug that has bound to the DNA. The *k*_*ij*_ parameters are rate constants that describe the movement of doxorubicin between each of these compartments; for example, *k*_*FE*_ describes the rate of drug transfer from the free, intracellular compartment to the extracellular compartment. Similar definitions apply to *k*_*EF*_ and *k*_*FB*_. The volumes of the intracellular and extracellular compartments are denoted with *v*_*I*_ and *v*_*E*_, respectively. The model is illustrated in Fig. 2a. Of note, each cell line is assumed to have a single set of compartment model parameters (i.e., *k*_*EF*_, *k*_*FE*_, and *k*_*FB*_), and those parameters are assumed to be independent of drug concentration and drug exposure time. Further, to simplify the model, saturation kinetics for doxorubicin transport are not explicitly included.

The extracellular and intracellular compartments were defined from the cell segmentation. To create fluorescent intensity timecourses for the intracellular and extracellular compartments, fluorescence signal was averaged within the respective (segmented) compartments on each image. These two intensity timecourses (extracellular and intracellular) were converted to concentration, as doxorubicin concentration is proportional to observed fluorescence intensity (Fig. 2d). The volume of the extracellular compartment, *v*_*E*_, was set to 250 µL, the volume of media in each well. The intracellular volume, *v*_*I*_, was estimated by multiplying the number of cells seeded (10,000) by an estimate of cell volume (an ellipsoid model was fit to cell segmentation results). A nonlinear least squares approach implemented in MATLAB (Natick, MA) was used to fit Eqs (1–3) to the concentration timecourses for each treatment condition to generate estimates for *k*_*EF*_, *k*_*FE*_, and *k*_*FB*_. Note that the extracellular compartment was treated as a well-defined, experimentally-controlled input function and was not fit by the model. For example, to generate the extracellular compartment timecourse illustrated in Fig. 2e, a bolus of doxorubicin was added to the experimental well *t* = 0 hours. At *t* = 6 hours, the drug was removed *via* media replacement; i.e., all drug-containing media is removed from the well, and fresh, drug-free media was added. This input function was used to perturb the system to measure the underlying cell line-specific compartment model parameters. The compartment modeling approach is outlined in Fig. 2, and details of the model fitting are included in Supplementary Materials.

### Treatment response monitoring

**Figure 3:**
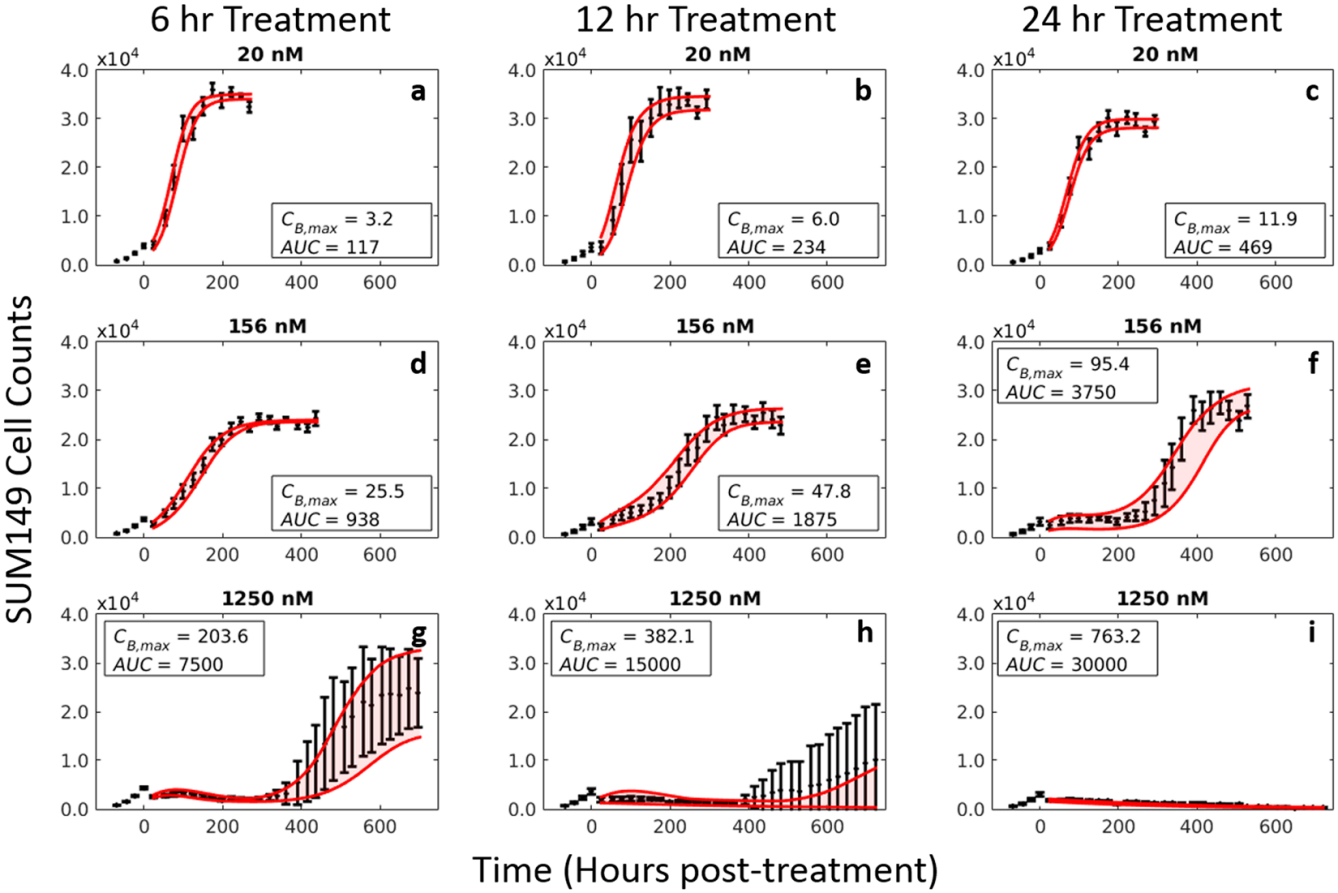
Impact of doxorubicin concentration and exposure time on response of SUM-149PT cells. The SUM-149PT cell line was plated and serially imaged *via* fluorescence microscopy for 30 days following time-resolved doxorubicin treatments. Nuclear counts from these images are displayed below in black with error bars representing the 95% CI from the six experimental replicates. These counts are fit to Eqs (4 and 5) as described Section 2.5. Model fits with 95% CI are superimposed on the cell counts. The SUM-149PT cell line demonstrated a graded dose-dependent and time-dependent response to doxorubicin treatment. At low concentrations, no appreciable treatment effect is noted regardless of exposure time (**a**–**c**). At higher concentrations and exposure times, the population growth rate slows (**d**,**e**), eventually demonstrating a prolonged response to therapy with subsequent regrowth of the population (**f**–**h**). At very high concentrations and exposure times, no population regrowth is observed (**i**).

**Figure 4:**
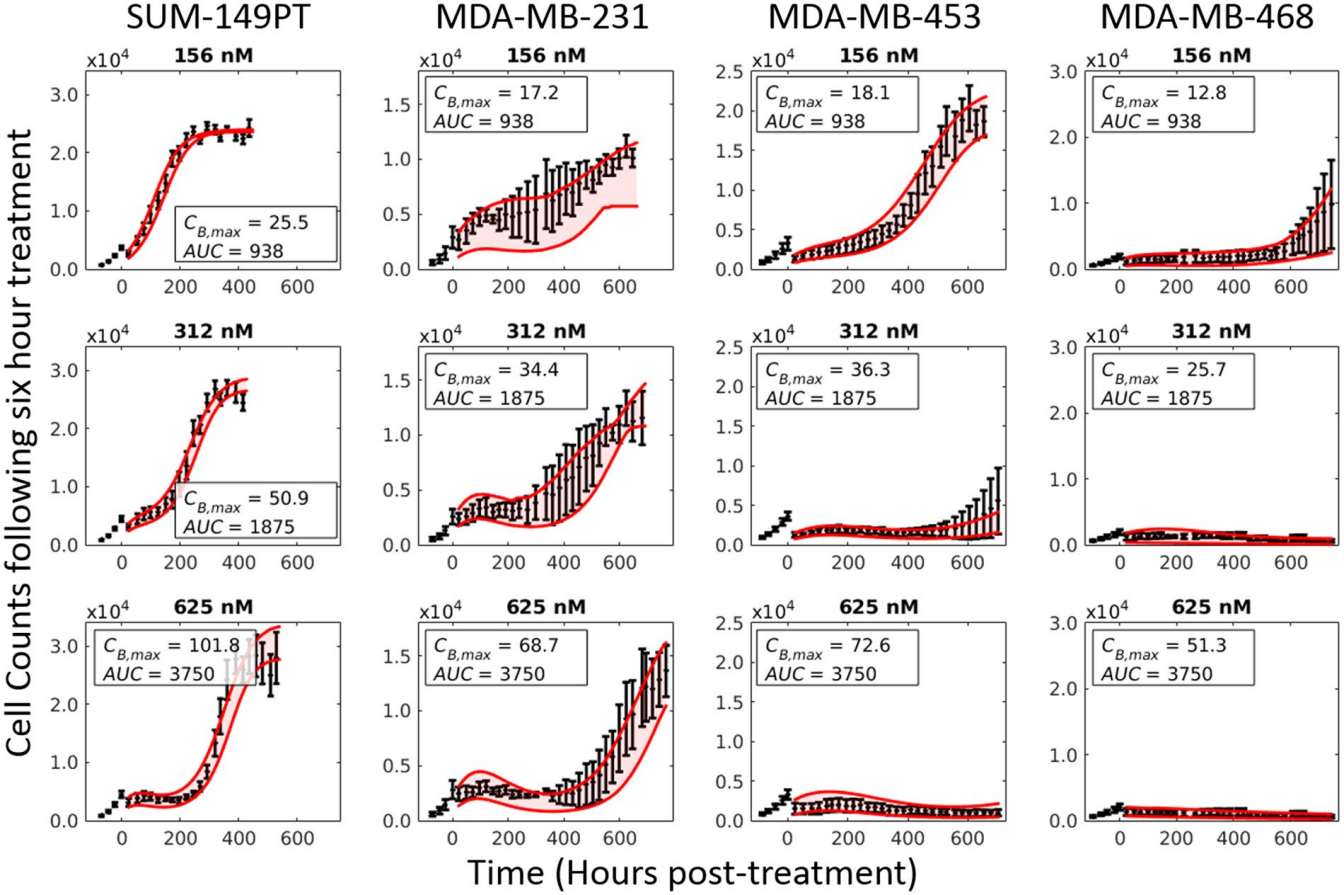
Dose-response curves in a panel of TNBC cell lines. Each cell line was plated and serially imaged *via* fluorescence microscopy for 30 days following a 6-hour doxorubicin treatment. Nuclear counts from these images are displayed below in black with error bars representing the 95% CI from the six experimental replicates. Each column corresponds to an individual cell line, and each row corresponds to a doxorubicin concentration. These counts are fit to Eqs (4–5) as described Section 2.5. Model fits with 95% CI are superimposed on the cell counts. While there is significant variability in cell line sensitivity to doxorubicin treatment, the dynamics of each cell line follows a similar pattern: following treatment the population growth rate slows as a function of treatment, and depending on the treatment duration and concentration, a rebound in population growth rate is observed.

Each H2B-labeled TNBC cell line was added to 96-well microtiter plates at ~2,500 cells per well. Cells were grown for at least three days to allow for a pre-treatment proliferation rate to be estimated. Doxorubicin was then introduced at concentrations ranging from 2500 to 10 nM with a two-fold dilution series and subsequently removed *via* media replacement after 6, 12, or 24 hours (areas under doxorubicin concentration-time curve ranging from 60 to 60000 nM∙hr). These experimental conditions were designed such that the areas under the doxorubicin curves overlapped those observed *in vivo* ^31^. These cells were imaged daily *via* fluorescent microscopy for at least 30 days following application of doxorubicin. For these treatment response studies, fluorescence microscopy images were collected using a Synentec Cellavista High End platform (SynenTec Bio Services, Münster, Germany) with a 20x objective and tiling of 21 images. Exposure times with 570 nm light were optimized for each cell line to account for varying label strength and ranged from 600–650 ms. Nuclei were segmented and counted in ImageJ (http://imagej.nih.gov/ij/) using a previously-described method^37^ to quantify cell population. Six replicates of each of the 30 treatment conditions (nine concentrations plus a control for each drug exposure time) were collected for each cell line. Media was refreshed every 3 days for the duration of each experiment to ensure sufficient growth conditions for surviving cells. Representative cell count data from these experiments are shown in Figs 3 and 4.

### Treatment response model

Doxorubicin canonically induces DNA damage by intercalating DNA bases, stabilizing the topoisomerase II complex, and inducing DNA damage *via* free radical formation^35^. At high doses (here, dose is defined as a summary statistic of a treatment condition, consolidating drug concentration and drug exposure time, and is denoted *D*), extensive DNA damage often results in cell death *via* apoptosis. Low to moderate doses of doxorubicin induce cell senescence, and cell death occurs primarily *via* mitotic catastrophe^38, 39^. Whereas apoptosis is immediate (on the order of hours to days), mitotic catastrophe is a relatively protracted process (on the order of several days). This is likely due to the fact that cells must progress through the cell cycle to reach mitosis for this mode of death to occur, and doxorubicin is known to cause cell cycle arrest. These processes were modeled by a logistic growth model, Eq. (4), modified by either one of two time-dependent response functions, Eq. (5A) and (B), reflecting the distinct forms of cell death, as follows:

4 $$\frac{d{N}_{TC}(t)}{dt}=({k}_{p}-{k}_{d}(t,D)){N}_{TC}(t)(1-\frac{{N}_{TC}(t)}{\theta (D)})$$

5A $${k}_{d}(t,D)=\{\begin{array}{cc}0 & t < 0\\ {k}_{d,A}(D) & t\ge 0\end{array}$$

5B $${k}_{d}(t,D)=\{\begin{array}{cc}0 & t < 0\\ {k}_{d,B}(D)r(D)t{e}^{1-r(D)t} & t\ge 0\end{array}$$

where *k*_*p*_ and *k*_*d*_ are the proliferation and dose-specific death rates, respectively, *r* is a dose-specific constant describing the rate at which treatment induces an effect, *θ* is the dose-specific carrying capacity describing the maximum number of cells that can be supported by the experimental system, and *N*_*TC*_(*t*) is the number of tumor cells at time *t*. Prior to treatment (i.e., *t* < 0), cells are modeled to have a constant proliferation rate, *k*_*p*_. Following treatment at *t* = 0, Eq. (5A), assumed an immediate transition from the pre-treatment growth rate to a stable, post-treatment rate. Eq. (5B), allowed for a smooth induction of drug effect following treatment, while ultimately allowing for recovery of the cell population. A weighted averaging approach, detailed below, was used to incorporate both Eq. (5A) and (B) in the treatment response model. Cell populations are assumed to be homogeneous in that the average behavior of the population is used to describe population dynamics. Of note, an analytic solution of Eqs (4 and 5) was derived to improve computational speed.

For each cell line, Eq. (4) was fit to pre-treatment and untreated control data, yielding a single, cell-line specific estimate for the proliferation rate, *k*_*p*_, and carrying capacity, *θ*. Fixing *k*_*p*_ for each cell line, the treatment response models, Eq. (5A) and (B), were then fit to the post-treatment data.

For each cell line, all data from a single doxorubicin exposure time experiment were considered simultaneously in the parameter optimization. Separate parameter estimates were made for each doxorubicin concentration in each exposure time dataset. Specifically, parameter estimates and the corresponding 95% confidence intervals were obtained for *k*_*d,A*_ and *θ* in Eq. (5A), and *k*_*d,B*_, *r*, and *θ* in Eq. (5B) from the post-treatment cell counts. To perform this estimation, a nonlinear least squares approach was implemented in MATLAB, utilizing the trust-region reflective algorithm. Notably, in fitting each model, a regularization term was introduced to the objective function, *G*(*x*), to penalize non-smooth variation in parameter values with respect to treatment conditions as follows:

$$\mathop{\min }\limits_{x}G(x)=\sum _{c={c}_{i}}^{{c}_{f}}(\sum _{t={t}_{i}}^{{t}_{f}}{(\frac{{Y}_{t,c}-{\hat{Y}}_{t,c}(x)}{{Y}_{t,c}})}^{2}+\alpha {D}_{c}{(x)}^{2}),$$

where *x* is the set of parameters, *Y*_*t,c*_ is the measured cell counts at time *t* and concentration *c*, *Ŷ*_*t,c*_(*x*) is the model-estimated cell counts at time *t* and concentration *c* when the model is evaluated with parameters *x*, *c*_*i*_ and *c*_*f*_ are the minimum and maximum drug concentrations respectively, *t*_*i*_ and *t*_*f*_ are the initial and final timepoints respectively, and *α* is an empirically-determined positive constant that weights the contribution of the regularization term, *D*_*c*_(*x*), which is a first derivative operator that estimates the local derivative of the parameters with respect to treatment condition (as described below). The regularization term provides structure to parameter estimates that are otherwise unable to be resolved with the treatment response data. In turn, the regularization term improves performance of the local regression approach used for predictions in Section 2.6, which is sensitive to local variance in parameter estimates. Details of the fitting approach are included in Supplementary Materials.

The maximum bound concentration of doxorubicin (*C*_*B,max*_) and the area under the curve of the extracellular concentration timecourse (*AUC*) were both used to summarize each treatment condition (*D* in Eqs (4 and 5))^35^. We hypothesized that the *C*_*B,max*_ metric would sufficiently describe both the topoisomerase-II mechanism of doxorubicin as well as doxorubicin’s free-radical mechanism, due to redox cycling of doxorubicin that persists within cells^40^. To calculate *C*_*B,max*_, the compartment model (i.e., Eqs (1–3)) was populated by cell-line-specific parameters and run forward in time using the specified extracellular concentration timecourse for each treatment condition. *C*_*B,max*_ was defined to be the maximal concentration in the bound compartment during the model evaluation. As doxorubicin is hypothesized to also have an extracellular effect^21, 41^, the *AUC* was also used as a descriptor of treatment condition. *AUC* was defined as the integral of the extracellular concentration timecourse with respect to time (simply (doxorubicin concentration) × (exposure time) in the pulsed treatments used in this study).

To generate a single best-fit model, a weighted averaging approach was employed. Model weights were calculated from the Akaike information criterion (AIC) for each *k*_*d*_(*t*) model (i.e., Eq. (5A) and (B))^42^. The AIC is a measure of model likelihood that balances goodness of fit with the number of free parameters. The AIC for model *i* can be calculated with the following equation:

$$AI{C}_{i}=n\,\mathrm{ln}\,\frac{RSS}{n}+2p,$$

where *n* is the number of data samples, *RSS* is the residual sum of squares of the fit-optimized model, and *p* is the number of model parameters. The normalized probability of model *i* being the best model, *w*_*i*_, among all proposed models can then be calculated:

$${w}_{i}=\frac{{e}^{-\frac{1}{2}{{\rm{\Delta }}}_{i}}}{\sum _{j=1}^{R}{e}^{-\frac{1}{2}{{\rm{\Delta }}}_{j}}},$$

where Δ_*i*_ is the difference in AIC values between model *i* and the model with the minimal AIC value and *R* is the total number of models^43, 44^. The best-fit model, *N*_*TC*_(*t*), can then be calculated by weighting Eqs (4 and 5) as follows:

$${N}_{TC}(t)={w}_{A}{N}_{TC,A}(t)+{w}_{B}{N}_{TC,B}(t),$$

where *N*_*TC*_,_*A*_(*t*) and *N*_*TC*_,_*B*_(*t*) are the solutions to Eq. (4) populated with Eq. (5A) and (B) respectively, and *w*_*A*_ and *w*_*B*_ are the respective weights for those models.

This fitting approach was validated on a synthetic dataset to ensure that parameter estimation routines successfully returned true model values (Supplementary Figs S4 and S5). To determine the effect of parameter variance on model behavior, the sensitivity of model predictions at the end of the experiment to each parameter was measured using the extended Fourier Amplitude Sensitivity Test^45^. The total-order sensitivity index, *S*_*TI*_, is reported. This metric is scaled from 0 to 1 and represents the fraction of model output variance that can be apportioned to variance in the parameter under investigation (Fig. S6, Supplementary Materials).

### Prediction of treatment response

The proposed model, as constructed, can accommodate a range of treatment times and concentrations. While this model is intended as a more general predictive framework, to demonstrate the utility of the modeling approach, the ability of the model to predict population changes following treatment at new concentrations and exposure times was evaluated. In this example, data from a single exposure time (12 hours; i.e., the ‘training set’) is used to train the model (i.e., Eqs (4 and 5)) to predict cell counts following treatments for 6 and 24 hours (i.e., the ‘test set’). This analysis was repeated using each exposure time dataset as a training set (e.g., 6- hour dataset used to predict cell counts following 12- and 24-hour treatments).

Model parameters and weights first were fit to the treatment response data in the training set as described in Section 2.5. Next, each treatment condition in the test set was described by its *C*_*B,max*_ and *AUC* values. As these values in the test set may not overlap exactly with those values in the training data, localized linear regression models were used to interpolate parameter space to generate parameter estimates at the specified *C*_*B,max*_ and *AUC* values^46^. This approach fits a linear model to training data near the *C*_*B,max*_ and *AUC* of interest. Model weights (i.e. *w*_*A*_ and *w*_*B*_) for the test set then were estimated through a binomial logistic model. This logistic function was trained to define the relationship between estimated model weights in the training set and the corresponding *C*_*B,max*_, *AUC*, and model parameter values. Finally, models were initialized with the first post-treatment measurements in the test set and run forward using the estimated parameter values to produce cell count predictions. This approach is outlined in Fig. S9 (Supplementary Materials).

The mean percent error across all timepoints and mean percent error at the end of the experiment are reported for the predicted models and corresponding best fit models. Confidence intervals on the predicted timecourses were constructed through a bootstrap analysis described in Supplementary Materials.

## Results

### Doxorubicin uptake

A three compartment model was sufficient to describe doxorubicin uptake in all cell lines. The mean percent errors of the model fit across all treatment conditions were 31.8%, 34.6%, 23.5%, and 26.8% for the SUM-149PT, MDA-MB-231, MDA-MB-453, and MDA-MB-468 cell lines respectively. Model residuals are shown in Fig. S2 (Supplementary Materials). A sample doxorubicin uptake curve is displayed in Fig. 2e along with compartment model parameter fits for each cell line with 95% confidence intervals. Significant differences were observed when comparing parameter fits from different cell lines. For example, *k*_*EF*_ is significantly greater in the MDA-MB-231 line than in the MDA-MB-468 line, indicating that doxorubicin diffuses more quickly into MDA-MB-231 cells (*p* < 0.05).

### Doxorubicin treatment response

Experimentally, all cell lines demonstrated a graded concentration-dependent and time-dependent response to doxorubicin treatment. Prior to treatment with doxorubicin at *t* = 0, each cell line displayed exponential growth. The proliferation rate (*k*_*p*_) of the SUM-149PT, MDA-MB-231, MDA-MB-453, and MDA-MB-468 cell lines were 2.69 × 10^−2^ hr^−1^, 2.23 × 10^−2^ hr^−1^, 1.64 × 10^−2^ hr^−1^, and 1.18 × 0^−2^ hr^−1^, respectively. In untreated controls, each cell line demonstrated logistic growth with cell-line specific carrying capacities (*θ*) of 3.81 × 10^4^, 1.86 × 10^4^, 2.21 × 10^4^, and 1.64 × 10^4^ for the SUM-149PT, MDA-MB-231, MDA-MB-453, and MDA-MB-468 cell lines (Fig. S3, Supplementary Materials).

Following treatment, responses varied from continued, positive growth up to immediate population regression. This spectrum of responses is illustrated by the SUM-149PT response data in Fig. 3. At low doses (*AUC* < 480 nM∙hr) doxorubicin has little effect, and cell populations continue to grow exponentially up to a carrying capacity (3a–c). As concentration and exposure time increase, the population growth rate appears to slow (3d). Eventually, a nonlinear response defined by a protracted slowing of population growth rate with a recovery back to pre-treatment growth rate is observed (3e–h). At high doses (*AUC* > 25 × 10^3^ nM∙hr), the cell population rapidly declines (*k*_*d*_,_*a*_ ≥ 2.9 × 10^−2^ hr^−1^), and no population rebound is observed during the experiment (3i).

The dose levels that correspond to the effects described above were specific to each cell line. In Fig. 4, cell counts from each cell line following doxorubicin treatment for six hours at three concentrations are shown. The SUM-149PT line is relatively insensitive to doxorubicin therapies, demonstrating continued growth in all treatment conditions shown in Fig. 4. Comparatively, the MDA-MB-468 line is very sensitive to doxorubicin therapy, demonstrating complete population regression at low doxorubicin doses (*AUC* ≥ 186 nM∙hr). The MDA-MB-231 and MDA-MB-453 cell lines displayed intermediate sensitivity. Despite the differential sensitivities, each of these cell lines followed the same general pattern described above.

### Model fits

As described in Section 2.5, the treatment response model was fit to each treatment condition. These model fits and 95% confidence intervals are superimposed on the cell counts in Figs 3 and 4. The mean percent error across all timepoints and mean percent error at the end of experiment (EoE) for the best-fit model to the SUM-149PT cell line after 6 and 24 hours of doxorubicin treatment are reported in Table 1. As shown in Table 1, the model was able to accurately capture a wide range of treatment conditions very accurately with mean percent errors of ≤15% for concentrations less than 625 nM after 6 hours of treatment. At higher concentrations, the model appears to perform poorly with mean errors >25%. However, in these cases, the small number of cells results in noisier measurements at all timepoints. Corresponding statistics for MDA-MB-231, MDA-MB-468, and MDA-MB-453 cell lines can be found in Table S1 (Supplementary Materials).

**Table 1:** Table of model statistics for SUM-149PT cell line following 6 and 24 hours of treatment with doxorubicin.

Concentration (nM)	SUM-149PT							
	6 hour Doxorubicin Treatment				24 hour Doxorubicin Treatment			
	Average % Error		Mean % Difference EoE		Average % Error		Mean % Difference EoE	
	Best-fit	Predicted	Best-fit	Predicted	Best-fit	Predicted	Best-fit	Predicted
10	5.3	5.5	3.8	4.7	6.4	7.1	5.7	5.7
20	5.3	5.6	3.0	3.6	5.6	9.5	4.3	7.0
39	6.1	6.6	4.6	6.3	5.5	10.2	4.9	8.1
78	6.1	7.1	5.0	6.6	10.3	10.5	8.7	9.1
156	4.6	5.3	4.5	4.0	22.7	22.5	12.0	9.9
312	9.4	13.6	6.8	5.9	31.4	32.5	25.2	26.9
625	15.0	16.9	13.1	11.9	50.8	53.6	120.3	150.9
1250	34.0	37.3	34.3	34.2	30.2	40.6	23.1	101.0
2500	24.4	42.5	61.1	159.9	40.0	59.0	32.0	117.3
Average Errors	12.2	15.6	15.1	26.3	22.5	27.3	26.2	48.4

**Figure 5:**
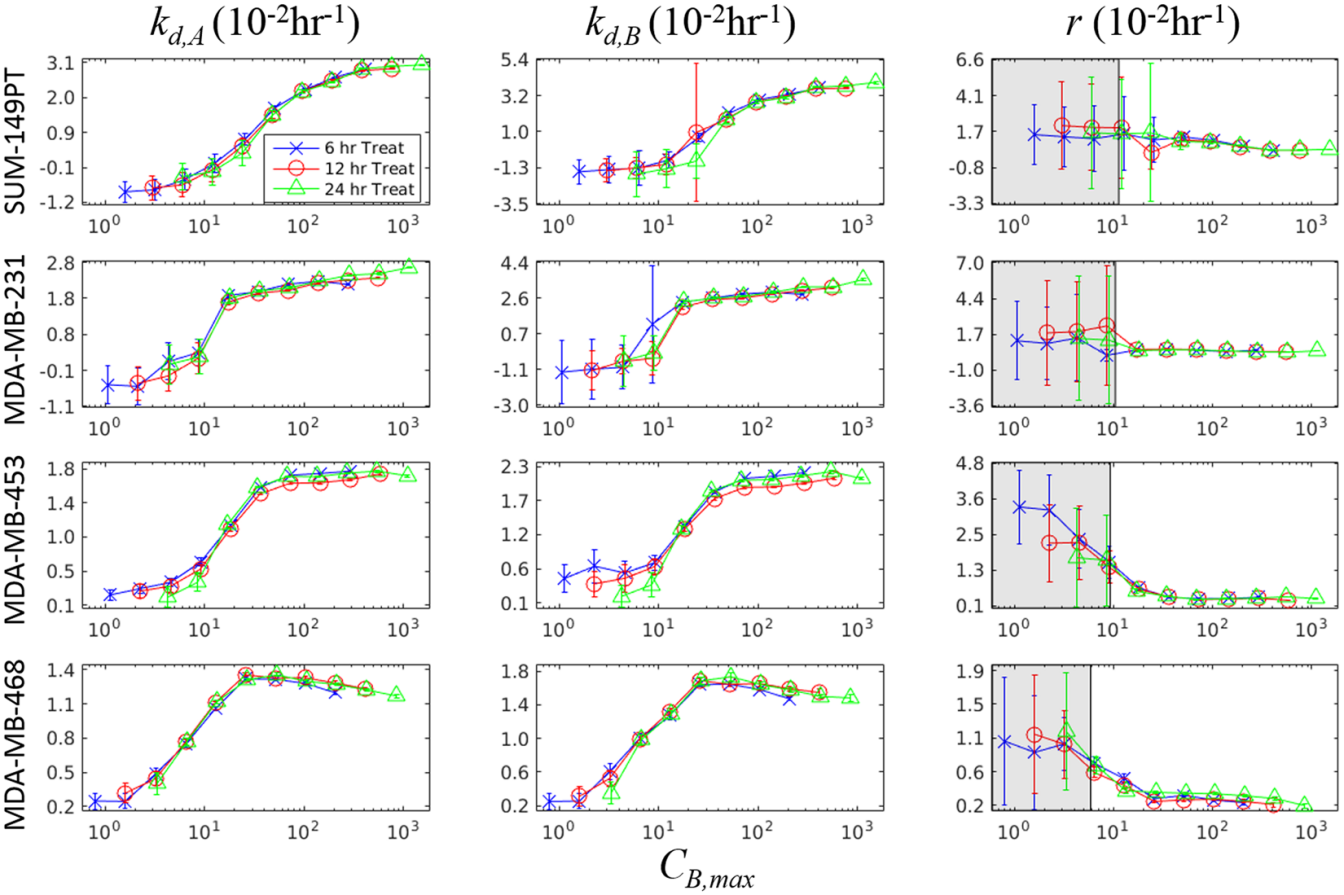
Parameter fits from Eq. (5) in a panel of TNBC cell lines as a function of *C*_*B,max*_. The parameters in Eq. (5) are fit to each treatment condition as described in Section 2.5 and plotted with 95% confidence intervals against the cell-line specific simulated *C*_*B,max*_ from Eqs (1–3). The blue X’s, red O’s, and green Δ’s represent the parameter fits extracted from the 6, 12, and 24 hour exposure time datasets respectively. Model parameters estimated from each exposure time appear to collapse on each other, when described by *C*_*B,max*_ – a summary statistic of each treatment condition. This indicates that the compartment model is effective at describing the treatments. Further, given that each cell line appears to follow a single trajectory for each parameter, this model can be used to predict cell population response to any predefined input function. The gray areas for parameter *r* represent treatment ranges where the total-order sensitivity index (*S*_*TI*_), which describes the effect of parameter variation on model prediction variation, is ≤0.3. Thus the large variance in parameter estimates here has a limited impact on model predictions.

Model parameter values changed with respect to treatment conditions within a given cell line. In Fig. 5, the parameter values with corresponding 95% confidence intervals extracted from experiments with the SUM-149PT cell line are reported. Note that the parameter values extracted across all exposure-time experiments for all investigated cell lines appear to collapse to a single curve for each parameter when plotted as a function of *C*_*B,max*_. Similarly, the carrying capacity (*θ*) appears to change slightly as a function of treatment condition (Fig. S7, Supplementary Materials). However, *θ* was unable to be estimated for high doxorubicin doses that induce population regression. Further, different models are selected over the range of treatments. Eq. (5B) is favored at lower *C*_*B,max*_ values (*w*_*B*_ ≈ 1) for the SUM-149PT cell line, while Eq. (5A) is selected at higher values (*w*_*B*_ ≈ 0) (Fig. S8, Supplementary Materials). Of note, the model is relatively insensitive to values of *r* at low *C*_*B,max*_ with *S*_*TI*_ ≤ 0.3 (meaning that other parameters account for 70% of model variation in this range).

### Model predictions

**Figure 6:**
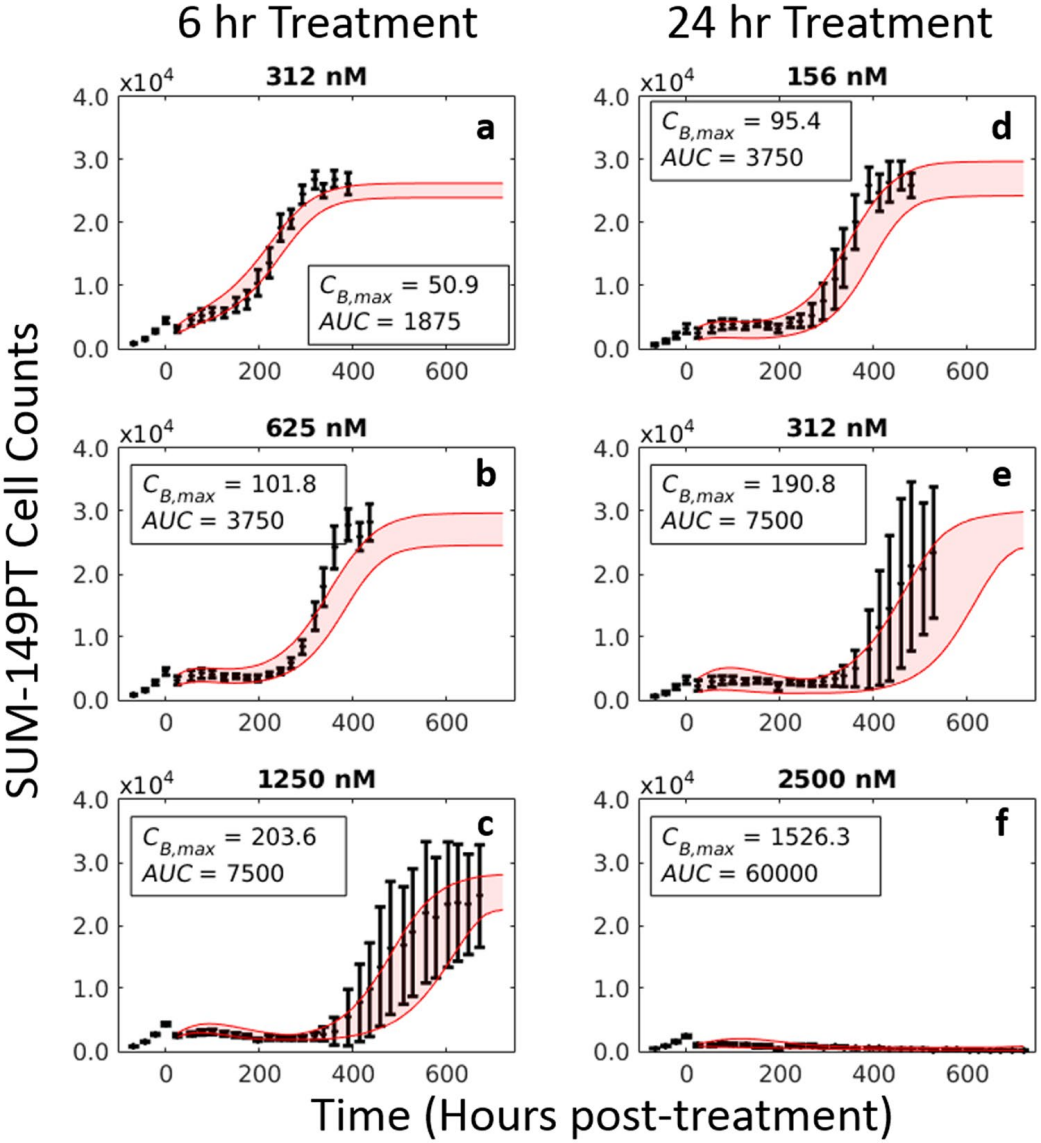
Model prediction results in SUM-149PT cell line. As described in Section 2.6, model parameters (*k*_*d,A*_, *θ* in Eq. (5A) and *k*_*d,B*_, *r*, *θ* in Eq. (5B)) were fit to each treatment condition in the training set (12-hour exposure dataset). These parameter fits were then described by local regression models to generate model parameter estimates for treatments in the test set (6- and 24-hour exposure datasets). Final predictions represent a weighted average of Eq. (5A) and (B), and a bootstrap analysis was used to generate a 95% confidence interval for these predictions (red overlay). A series of predictions in the SUM-149PT cell line following 6- and 24-hour doxorubicin treatments at three doxorubicin concentrations are shown. Nuclear counts from these experiments are displayed in black with error bars representing the 95% CI from the six experimental replicates. Each column corresponds to an exposure time. The response of a TNBC cell line can be predicted using experimentally-derived PK and PD parameters.

The prediction scheme in Section 2.6 was trained on the 12-hour exposure time dataset in the SUM−149PT cell line to generate predictions of population dynamics following 6- and 24-hour doxorubicin treatments. A set of model predictions is shown in Fig. 6 overlaid on experimental data, and predictions appear to qualitatively match experimental data. Table 1 reports the mean percent error across all timepoints and mean percent error at the EoE of the predictions for the SUM-149PT cell line at each concentration. As shown in Table 1, the error rates of the predicted model compare favorably to those of the best fit model, with the average percent error differing by 3.4% between the groups, on average. Further, the predictive model performs very well according to average error at concentrations up to 625 nM with an average error of 8.7% across those concentrations. The predictions degrade along with the best-fit model at higher concentrations. Similar results were obtained when the prediction scheme was trained with the 6-hour and 24-hour datasets (Tables S2 and S3 respectively, Supplementary Materials). The average percent error differed by 4.1% between the best fit and predicted models on average in these experiments.

## Discussion

A modeling approach has been introduced that can be used to summarize the PK/PD properties of doxorubicin in TNBC cell lines. Cell-line specific model parameters can be estimated from experimental data, revealing phenotypic heterogeneity in PK/PD properties not previously quantified. Further, the PD properties were found to vary as a function of *C*_*B,max*_ and *AUC*, summary statistics of doxorubicin PK. This allows for accurate prediction of cell population behavior for up to one month following prescribed doxorubicin treatments *in vitro*.

The pharmacokinetics of doxorubicin binding in a panel of cell lines can be characterized by a three compartment model. Similar to findings by Shin *et al*., there are significant differences in doxorubicin pharmacokinetics among TNBC cell lines^32^. Interestingly, these parameters are only loosely correlated with response. For example, the MDA-MB-231 has a greater uptake of doxorubicin (as estimated by *C*_*B,max*_) than the MDA-MB-468 line; however, the MDA-MB-468 line is more sensitive to doxorubicin therapy. This suggests that each cell line has an intrinsic sensitivity to stress by doxorubicin. More generally, this model can be leveraged to isolate and normalize for variable uptake dynamics in the context of doxorubicin resistance. This could help refine approaches to identify mechanisms of resistance and subsequently develop targeted agents to address those mechanisms.

The model relating treatment variables (concentration and duration) to subsequent cell population size dynamics proposed in this work captures behavior across a range of TNBC cell lines. While each cell line can be described by a specific set of parameters, there is an underlying behavior common to all cell lines that is described by the model: an apparent continuum of responses from exponential growth to population regression as doxorubicin concentration and exposure time are increased. Further, TNBC response to doxorubicin therapy generally appears to be a deterministic process. Over a wide range of treatment conditions, cell populations responded consistently, as evidenced by the confidence intervals on the cell count data in Figs 3, 4 and 6, the overlapping parameter curves in Fig. 5, and the accuracy of predictions in Table 1. Several models in the literature have assumed a direct relationship between treatment variables and cellular response – either immediate^47^ or following some fixed delay^22, 48^. Consistent with those delay models, the data presented in this work indicate that drug effects occur on a slower timescale relative to drug binding. Characterizing and reporting on these dynamic measures would enhance information from traditional potency-based assays. Understanding the dynamics of therapeutic administration and treatment response can inform drug treatment schedules and will provide guidance to optimize response monitoring.

Interestingly, there appears to be an upper threshold on doxorubicin treatment above which all cells die. As that concentration threshold is approached, increased variance is observed in population dynamics, especially at later timepoints. For example, in this range of therapy, one or two experimental replicates would regrow while no growth was observed in the other replicates (e.g., Fig. 3h). This contributes partly to the high error rates at high concentrations in Table 1. In these cases, the heterogeneity of the cell population or stochastic cell fate decisions may likely have an increased influence on population dynamics^49^. We emphasize that such increases in variance are more often observed as this treatment threshold is approached. This observation questions the use of maximum tolerated dosing schemes, which operate in this high-variance range^50, 51^. Considering the data presented in this work in the context of proposed adaptive dosing and metronomic dosing approaches^8–11^, there may exist a framework in which drug schedules can be customized for each patient to generate predictable changes in tumors. Indeed, the PK/PD modeling framework proposed in this work provides a means to more precisely test those alternative therapeutic approaches. Even in the current state of TNBC therapy, doxorubicin is often delivered on a predefined schedule for all patients with only doses adjusted for patient body-surface area. The demonstrated heterogeneity among TNBC cell lines, both in their uptake of doxorubicin and the effect of doxorubicin on those cell behaviors, suggests that additional metrics are needed for proper dosing of doxorubicin in TNBC. Tumor-specific PK properties may be required to normalize tumor response measurements to delivered doxorubicin dose.

This work is further distinguished through its use of a model averaging approach; i.e., the best-fit PD model is a weighted average of two distinct treatment response models (Eq. (5A) and (B)). Fundamentally, different cellular processes dominate over the dose range investigated (apoptosis at high doses, mitotic catastrophe at low to intermediate doses)^38, 39^. These disparate behaviors are observed in the data, and the model was constructed to account for these behaviors. Notably, Eq. (5A) is unable to explain the regrowth seen at low doses, and Eq. (5B) is unable to describe permanent population regression seen at high doses. The model averaging approach demonstrated here can be used to summarize the behavior of cell populations over the entire range of doses investigated. Further, this approach can be used to gain biological insight into the behavior of cell lines. Apoptosis is commonly treated as a switch-like process, which commits a cell to death at some biologically-defined threshold^52^. Similarly, a switch in model weights towards Eq. (5A) (apoptosis) is observed for each cell line as doxorubicin dose increases (Fig. S8, Supplementary Materials). Model averaging approaches can limit the insight gained from modeling as different models can be selected over the range of experimental conditions without an apparent pattern. However, explicitly incorporating biologically-motivated models into a model averaging framework may improve both model accuracy and expand the insight derived from modeling approaches.

As demonstrated in Fig. 6 and Table 1, the prediction framework proposed in this paper performs well across the range of treatments and cell lines investigated. This predictive modeling framework is dependent on: (1) the observation that model parameters are functions of treatment variables, and (2) these treatment variables can be summarized by *C*_*B,max*_ and *AUC*. Despite the relative simplicity of the models proposed in this work and the pharmacokinetic features used to predict parameter values, this framework is able to generate relatively accurate predictions to all experimental treatments in the SUM-149PT cell line, regardless of the training set used. While doxorubicin has been in clinical use for several decades, to our knowledge, measurements of its cellular effects have not previously been coupled to intracellular concentrations in a predictive framework. More broadly, this framework is readily amendable to predict response to other cytotoxic therapies. Although it is nearly certain that other cytotoxic therapies will require different parameter sets or, even, mathematical models, the coupled experimental-modeling approach presented in this work can be used to generate predictions following construction of those drug-specific models.

While the results of this study are promising, several limitations exist in the current approach. With respect to the compartment model proposed to describe doxorubicin pharmacokinetics, model parameters may change as a function of treatment concentration and duration, as suggested by the distribution of residuals seen in Fig. S2 (Supplementary Materials). Characterization of such variation through more extensive experiments may be possible, but doxorubicin exerts an effect on the cells over the course of the experiments – inherently changing the values of the compartment model. For example, cell size was observed to shrink during doxorubicin exposure. This reduced volume would enhance the fluorescent signal measured from intracellular space in these experiments. While the compartment model explicitly incorporated the volume of these compartments with estimates of cell volume, additional parameters would be needed to account for the time-dependent variation in compartment volumes. Indeed, when each treatment condition in the compartment modeling experiment was fit independently, the value of *k*_*FB*_ appeared to increase with concentration and duration of therapy (data not shown). While no significant violations of model assumptions are seen, it is difficult to rigorously test the assumption that parameters are independent of concentration and exposure time in the current dataset, which only contains four doxorubicin concentrations and two exposure times. Further, heterogeneity in the uptake of doxorubicin was observed. Within the field of view of the experiment, variation was noted from one cell to the next (Fig. 2c). As the modeling approach collapsed all cells into a single drug uptake timecourse, this heterogeneity was not considered. It would be of interest to track these cells over time to determine cell-specific parameters in relation to drug administration^53^. Further, this model does not explicitly include saturation kinetics for doxorubicin transport, which may contribute to the observed error rates. However, incorporating this heterogeneity would significantly increase the complexity of the proposed model, requiring additional equations and additional experimental data to describe each compartment model rate. Despite these limiting assumptions, the *C*_*B,max*_ term calculated with the three compartment model allowed for prediction of pharmacodynamic properties.

The treatment response model was inspired by observations of treatment response in these cell lines. While the treatment conditions were designed to replicate those observed *in vivo*, it remains unknown how the proposed model would respond to more complex treatment curves; e.g., biexponential decay curves observed *in vivo* ^31^. Such dynamics should, in theory, be captured by the proposed doxorubicin PK model, but work remains to validate that assumption. Application of the model to an *in vivo* system will also require spatial considerations. For example, significant heterogeneity in perfusion exists within a tumor, impacting both tumor growth and drug delivery^54^. The variable and immature vasculature may induce local microenvironmental changes (hypoxia, acidic extracellular pH) that alter the response to therapy^55, 56^. This modeling framework may need to be expanded to account for such spatial heterogeneity which can be characterized by (for example) quantitative imaging data^57^. However, the translation of the logistic growth formulation has already been realized in several *in vivo* models^58–62^.

In summary, these time-resolved treatment protocols sought to replicate the clinically observed pharmacokinetics of doxorubicin therapy more closely than the constant concentrations in previous dose-response assays. The proposed model, initialized with cell-line specific parameters, can describe the response to doxorubicin across a range of TNBC cell lines and treatment conditions. Further, within each cell line, the behavior collapses into a single path through parameter space as a function of treatment conditions. This observation allows for the *in vitro* response of each cell line to doxorubicin treatment to be predicted. Through the development of a mathematical model that explicitly considers both doxorubicin pharmacokinetics and pharmacodynamics, exploration of a wide range of treatment protocols that would be intractable experimentally is now possible. Specifically, this model provides an imminently scalable approach to predicting tumor changes in response to doxorubicin pharmacokinetics *in vivo*. This approach should allow for further refinement of biological models of doxorubicin treatment response, scalable predictions of tumor response in animal models, and, eventually, personalized, computationally-optimized treatment regimens that maximize tumor control with doxorubicin.

## Electronic supplementary material

Supplementary Materials
