# Supplementary material for: A Predictive Mathematical Modeling Approach for the Study of Doxorubicin Treatment in Triple Negative Breast Cancer
Source: Sci Rep. 2017 Jul 18;7:5725. doi: 10.1038/s41598-017-05902-z (PMC5516013; doi:10.1038/s41598-017-05902-z)
Supplement: Supplementary file 1 — Supplementary Materials [file 41598_2017_5902_MOESM1_ESM.pdf]

Supplementary Materials for:

A Predictive Mathematical Modeling Approach for the Study of Doxorubicin Treatment in Triple Negative Breast Cancer

Matthew T. McKenna, Jared A. Weis, Stephanie L. Barnes, Darren R. Tyson, Michael I. Miga, Vito Quaranta, Thomas E. Yankeelov

Cell culture

All cell lines were obtained through American Type Culture Collection (ATCC, http://www.atcc.org) and maintained in culture according to ATCC recommendations. All cell lines were tested after thawing for mycoplasma using a PCR-based method (MycoAlert, Lonza, Allendale, NJ), and any positive cultures were immediately discarded. To facilitate automated image analysis for identifying and quantifying individual nuclei in the time-lapsed fluorescent microscopy experiments (described below), each of the four cell lines was modified to express a histone H2B conjugated to monomeric red fluorescent protein (H2BmRFP; Addgene Plasmid 18982) as previously described[1–3]. Briefly, this plasmid was transduced into each cell line with replication-incompetent lentiviral particles. Following transduction, each cell line was sorted via flow cytometry by label intensity to collect the brightest 20% of the respective cell populations. Cell lines were passaged no more than 30 times before being discarded.

Doxorubicin image processing

Digital images were segmented into extracellular and intracellular compartments through a hybrid, semi-automated process. Prior to doxorubicin application, segmentation was performed exclusively on the brightfield images to identify cell boundaries. For each cell line, a single cell image was manually cropped, and the normalized cross-correlation was calculated between that cell and the entire image. Local maxima of the cross correlation were used to identify cells. The images were then manually reviewed to remove any false positives and to ensure all cells were identified. The initial cell segmentation mask that consisted of circles centered on each cell identified *via* cross-correlation were refined through an active contour model (Fig. 2b).

Following application of doxorubicin, segmentation was performed on the fluorescent images with a threshold-based approach. Briefly, the background was subtracted by taking the difference between the image and a cell-free well with the same doxorubicin concentration. An

adaptive histogram approach was used to enhance the contrast of this difference image, and the image was finally de-noised with a Wiener filter. The threshold was automatically selected for each image with Otsu's method, and this threshold was applied to generate the segmentation mask. The mask was refined using an active contour model (Fig. 2c). Finally, the mask was overlaid on brightfield images, and the segmentation result was manually reviewed.

Doxorubicin compartment modeling

Doxorubicin is thought to enter cells *via* diffusion, possibly through a saturable carrier-mediated process[4,5]. Once in the cell, doxorubicin is translocated to the nucleus where it intercalates DNA and stabilizes the topoisomerase II complex[6,7]. Doxorubicin may also be actively effluxed from the cell *via* p-glycoprotein[8]. Three compartment models of varying complexity were proposed to describe doxorubicin pharmacokinetics. The models are illustrated in Fig. S1. The model used in this work (Fig. 2a, Fig. S1b, Eqs. (1) – (3)) was selected because it best balanced model parameter parsimony with goodness of fit as measured by the Akaike information criterion[9].

A nonlinear least squares approach implemented in MATLAB was used to fit Eqs. (1) - (3) to the concentration timecourses for each treatment condition to generate estimates for k_{EF}, k_{FE}, and k_{FB}. Of note, the extracellular compartment was treated as a well-defined, experimentally-controlled input function and is not fit by the model. This input function is used to perturb the system to measure the underlying cell line-specific compartment model parameters. As model parameters were assumed to be invariant of doxorubicin concentration and exposure time, all treatment conditions were fit simultaneously to yield a single estimate of k_{EF}, k_{FE}, and k_{FB} for each cell line. The area under the doxorubicin curve in the extracellular compartment ($AUC_{c,t}$) was introduced to the objective function, $G(x)$, to normalize for treatment conditions as follows:

$$\min_x G(x) = \sum_{c=c_i}^{c_f} \left(\sum_{t=t_i}^{t_f} \frac{\left(Y_{t,c} - \hat{Y}_{t,c}(x)\right)^2}{AUC_{c,t}} \right)$$

where x is the set of parameters, $Y_{t,c}$ is the measured concentration at time t and concentration c, $\hat{Y}_{t,c}(x)$ is the model-estimated concentration at time t and concentration c when the model is evaluated with parameters x, c_i and c_f are the minimum and maximum drug concentrations respectively, and t_i and t_f are the initial and final timepoints, respectively. Without AUC

normalization, the residuals at high concentrations and exposure times would drive model fits at the expense of lower concentrations and exposure times. Model fit residuals are reported in Fig. S2.

Treatment response model fitting

The treatment response model was fit using a nonlinear least squares approach implemented in MATLAB, utilizing the trust-region reflective algorithm. Notably, in fitting each model, a regularization term was introduced to the objective function, $G(x)$, to penalize non-smooth variation in parameter values with respect to treatment conditions as follows:

$$\min_x G(x) = \sum_{c=c_i}^{c_f} \left(\sum_{t=t_i}^{t_f} \left(\frac{Y_{t,c} - \hat{Y}_{t,c}(x)}{Y_{t,c}} \right)^2 + \alpha D_c(x)^2 \right),$$

where x is the set of parameters, $Y_{t,c}$ is the measured cell counts at time t and concentration c, $\hat{Y}_{t,c}(x)$ is the model-estimated cell counts at time t and concentration c when the model is evaluated with parameters x, c_i and c_f are the minimum and maximum drug concentrations respectively, t_i and t_f are the initial and final timepoints respectively, and α is an empirically-determined positive constant that weights the contribution of the regularization term, $D_c(x)$, which is a first derivative operator that estimates the local derivative of the parameters with respect to treatment conditions as described by $C_{B,max}$ and AUC. $C_{B,max}$ is the maximum bound concentration of doxorubicin as estimated with Eqs. (1) – (3), and AUC is the area under the curve of the extracellular concentration timecourse. Notably, separate parameter estimates were made for each doxorubicin concentration in each exposure time dataset (i.e., each treatment condition). The regularization term provides an empirically-weighted constraint that encourages model parameters to vary smoothly a function of treatment condition (as described by $C_{B,max}$ and AUC). The model was initialized with the cell count at the timepoint following treatment for each individual replicate, and all subsequent timepoints were considered in the model fit. To avoid local minima, the fitting process was initialized with 50 sets of parameter estimates selected randomly from expected distributions for each parameter. k_d values were initialized by sampling a uniform distribution from [-2k_p, 5k_p]. r was bounded within [0.001, 0.05] hr^{-1}. These bounds correspond to observing the maximal death rate at 1000 and 20 hours respectively, reflecting the duration of the

experiment (~700 hours) and the sampling rate (24 hours). The experiments, as constructed, are unable to resolve estimates beyond these boundaries. Further, θ was bounded between [0.7θ, 1.3θ], where θ is the carrying capacity observed in the control data for each cell line. Notably, θ could not be resolved at high doses that induced population regression. In these cases, in which population size is much lower than θ, the logistic growth term has little influence on model behavior.

A cross-validation approach was used to tune the regularization parameter α. For each cell line, the other three cell lines were used as a training set to determine α for the held-out cell line. Each model (i.e., Eqs. (5A) – (5B)) was fit to each cell line in the training set using α values uniformly sampled from 0 to 10. The prediction scheme outlined in Section 2.6 was then run for each cell line. The α value yielding the best predictions as measured by mean percent error across the cell lines in the training set, were used in fitting the held-out cell line. The α values used to generate the fits in Figs. 3-5 were (α for Eq. (5A), α for Eq. (5B)): (10,1) for SUM-149PT, (6,1) for MDA-MB-231, (6,2) for MDA-MB-453, and (4,3) for MDA-MB-468.

Confidence intervals on the best-fit timecourses were constructed through a bootstrap approach. The six replicates for each treatment condition were resampled with replacement, and the fitting process described above was used to generate timecourses for each resampled dataset. This resampling was repeated 500 times, and the 95% confidence intervals on these fits were recorded.

This fitting approach was validated on a synthetic dataset to ensure that parameter estimation routines successfully returned true model values. The dataset was constructed through forward evaluations of the proposed models with five levels of additive noise drawn from a normal distribution with a standard deviations of 0%, 5%, 10%, 15%, and 20% of the simulated cell count (Figs. S4 and S5). Notably, the cell counting technique employed in this work has been shown to be highly accurate; when compared to manual counting, the automated counts are highly correlated ($R^2 = 0.99$)[10]. 22,500 simulations were run under each noise condition. High variance in parameter estimates was observed as values of r approached 0.05 hr^{-1} and values of $k_{d,B}$ approached 0 hr^{-1}. There exists intrinsic uncertainty at this limit as those rapid dynamics coupled with small $k_{d,B}$ effects cannot be resolved by the current data. This uncertainty in r for small $k_{d,B}$ does not affect model predictions as demonstrated by the sensitivity analysis in Fig. S6.

Prediction of treatment response

Confidence intervals on the predicted timecourses were constructed through a bootstrap analysis in which the six replicates for each treatment condition in the training set were resampled with replacement. This resampled training set was used to generate predictions. The resampling procedure was repeated 500 times, and the 95% confidence intervals on these predictions were computed.

References

1. Harris, L. A. *et al.* An unbiased metric of antiproliferative drug effect in vitro. *Nat. Methods* **13,** 497–500 (2016).
2. Tyson, D. R., Garbett, S. P., Frick, P. L. & Quaranta, V. Fractional proliferation: a method to deconvolve cell population dynamics from single-cell data. *Nature Methods* **9,** 923–928 (2012).
3. Quaranta, V. *et al.* Trait variability of cancer cells quantified by high-content automated microscopy of single cells. *Methods Enzymol.* **467,** 23–57 (2009).
4. Shin, K., Klosterhoff, B. S. & Han, B. Characterization of cell-type-specific drug transport and resistance of breast cancers using tumor-microenvironment-on-chip. *Mol. Pharm.* **13,** 2214–2223 (2016).
5. Nagasawa, K., Natazuka, T., Nomiyama, M., Ohnishi, N. & Yokoyama, T. Transport mechanism of anthracycline derivatives in human leukemia cell lines: uptake and efflux of daunorubicin and doxorubicin in HL60 and its resistant cells and comparison with those of pirarubicin. *Biol Pharm Bull* **19,** 100–105 (1996).
6. Tacar, O., Sriamornsak, P. & Dass, C. R. Doxorubicin: an update on anticancer molecular action, toxicity and novel drug delivery systems. *J. Pharm. Pharmacol.* **65,** 157–170 (2013).
7. Gewirtz, D. A. A critical evaluation of the mechanisms of action proposed for the antitumor effects of the anthracycline antibiotics adriamycin and daunorubicin. *Biochem. Pharmacol.* **57,** 727–741 (1999).
8. Shen, F. *et al.* Quantitation of Doxorubicin Uptake, Efflux, and Modulation of Multidrug Resistance (MDR) in MDR Human Cancer Cells. *J. Pharmacol. Exp. Ther.* **324,** 95–102 (2008).
9. Bozdogan, H. Model selection and Akaike's Information Criterion (AIC): The general theory and its analytical extensions. *Psychometrika* **52,** 345–370 (1987).
10. Frick, P. L., Paudel, B. B., Tyson, D. R. & Quaranta, V. Quantifying heterogeneity and dynamics of clonal fitness in response to perturbation. *J. Cell. Physiol.* **230,** 1403–1412 (2015).
11. Marino, S., Hogue, I. B., Ray, C. J. & Kirschner, D. E. A methodology for performing global uncertainty and sensitivity analysis in systems biology. *J. Theor. Biol.* **254,** 178–196 (2008).

Figure S1: Compartment model selection. Each of the models (a-c) were fit to the concentration timecourses as described in Section 2.3. The Akaike information criterion (AIC) was calculated for each of the models. The AIC is a measure of model likelihood that balances goodness of fit with the number of free parameters. Smaller AIC values indicate a more likely model. Model b is more likely than Model a for all cell lines. Model c results in a marginal improvement on Model b in two of the cell lines. Model b was selected to describe doxorubicin pharmacokinetics.

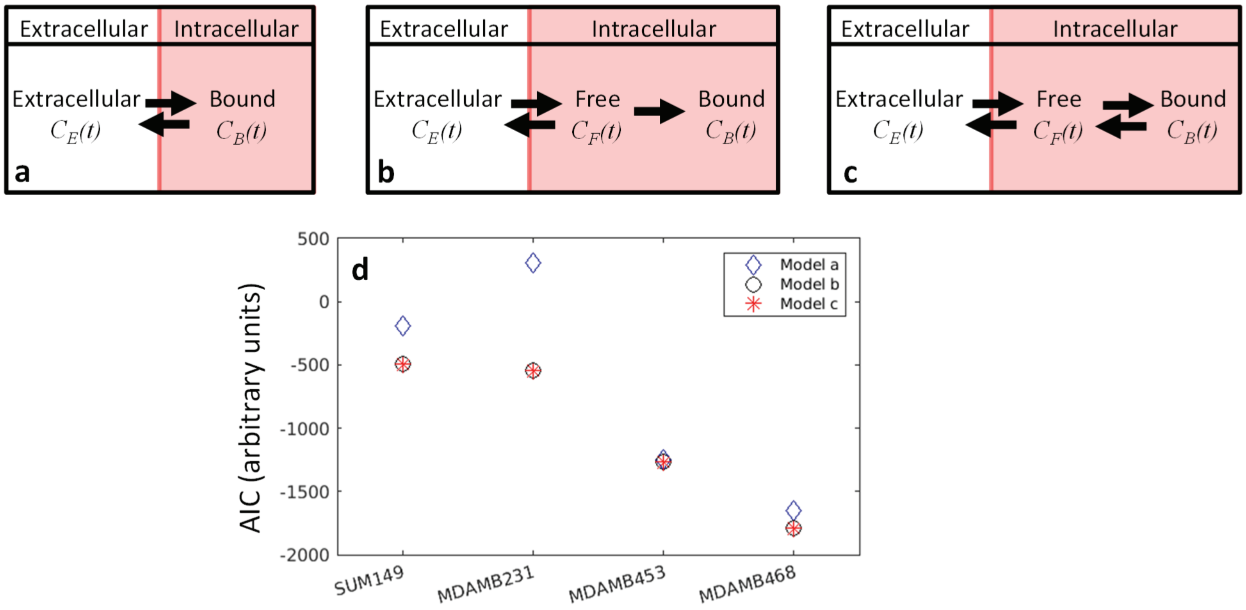

Figure S2: Residuals from compartment model fit. The doxorubicin concentration timecourses are fit to Eqs. (1) – (3) as described Section 2.3 and in Supplementary Materials. Residuals, normalized by the area under the doxorubicin curve in the extracellular compartment (AUC), are shown below. Each column corresponds to an individual cell line and each row to a given drug concentration. The red and black points represent residuals from the 6-hour and 12-hour exposure time datasets, respectively. While no significant violations of model assumptions are seen, it is difficult to rigorously test the assumption that parameters are independent of concentration and exposure time in the current dataset, which only contains four doxorubicin concentrations and two exposure times. Notably, higher errors are observed at low doxorubicin concentrations and low exposure times. In these cases, the low concentrations of doxorubicin in the intracellular compartment result in a low signal to noise ratio of the measurements.

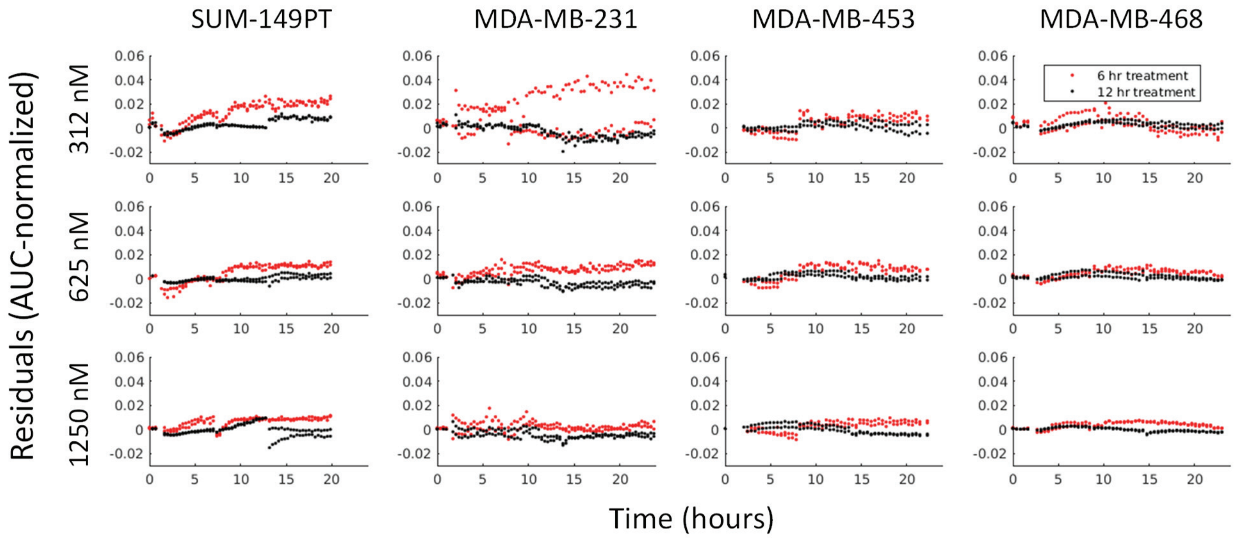

Figure S3: Control data from dose-response experiments in a panel of TNBC cell lines. Each cell line was plated and serially imaged *via* fluorescence microscopy for 30 days. Nuclear counts from these images are displayed below in black with error bars representing the 95% CI from the six experimental replicates. Each column corresponds to an individual cell line. Data are truncated when cell populations reached carrying capacity. These counts are fit to Eq. (4) with $k_d = 0$ as described Section 2.5. Model fits with 95% CI are superimposed on the cell counts. Each cell line demonstrates a characteristic growth rate (k_p) and carrying capacity (θ).

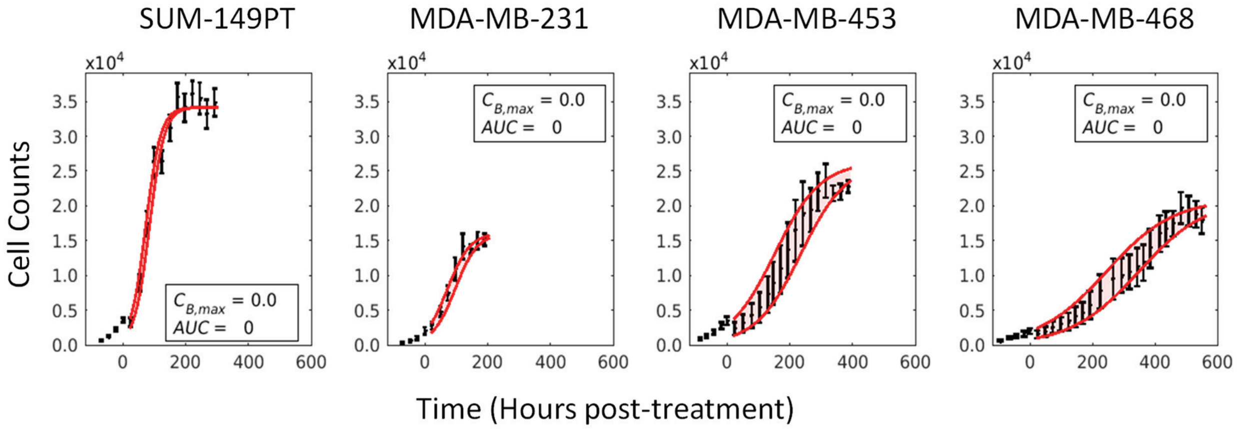

Figure S4: Model 5A was simulated with five levels of additive noise (0, 5, 10, 15, 20%). Parameters were recovered from those simulations, and the fit parameter value ($k_{d,A}$) is compared to its true value. The average value and 95% confidence interval of parameter fits are shown below. Thus, the fitting procedure can recover parameter values across all simulated conditions.

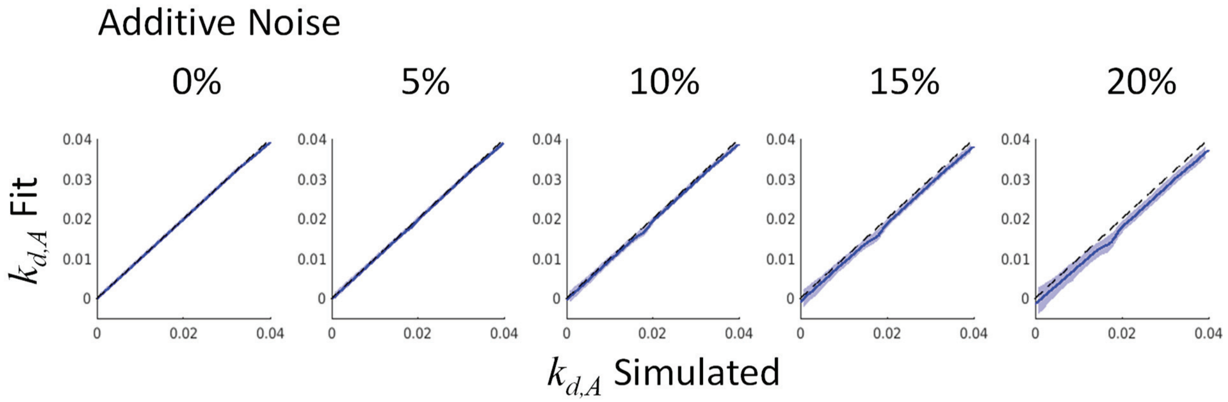

Figure S5: Model 5B was simulated with five levels of additive noise (0, 5, 10, 15, 20%). Parameters were recovered from those simulations, and the relative percent error of the fit parameters ($k_{d,B}$ and r) are reported. While the fitting procedure can recover parameter values across all simulated conditions, consistently high error rates in r estimates were observed at low values of $k_{d,B}$. The uncertainty in r for small $k_{d,B}$ does not affect model predictions as demonstrated by the sensitivity analysis in Fig. S6.

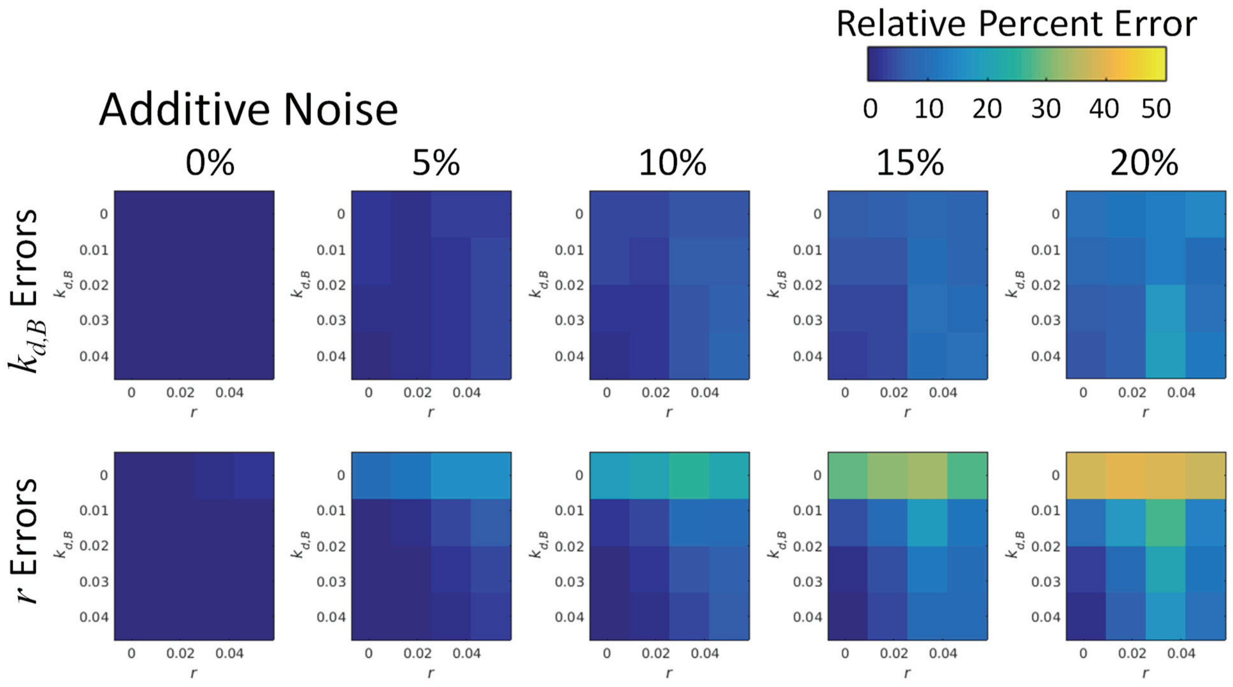

Figure S6: The sensitivity of model behavior to parameter variation was measured *via* the extended Fourier Amplitude Sensitivity Test (eFAST[11]) and reported with S_{TI}, the total-order sensitivity index. S_{TI} represents the fraction of model output variance that can be apportioned to variance in the parameter under investigation and is scaled from 0 (insensitive) to 1 (sensitive). The sensitivity of model behavior 7, 14, and 28 days following treatment are reported in (a-c) as a function of $k_{d,B}$. The errorbars represent one standard deviation. A *dummy* parameter is included for reference. This parameter is not in the model and thus the model should be insensitive to its variation. Notably, model behavior is insensitive to r for low values of $k_{d,B}$. Additionally, model behavior is insensitive to θ at early timepoints and high $k_{d,B}$ values as the cells counts are much lower than the carrying capacity in these conditions, limiting the effect of the logistic growth term.

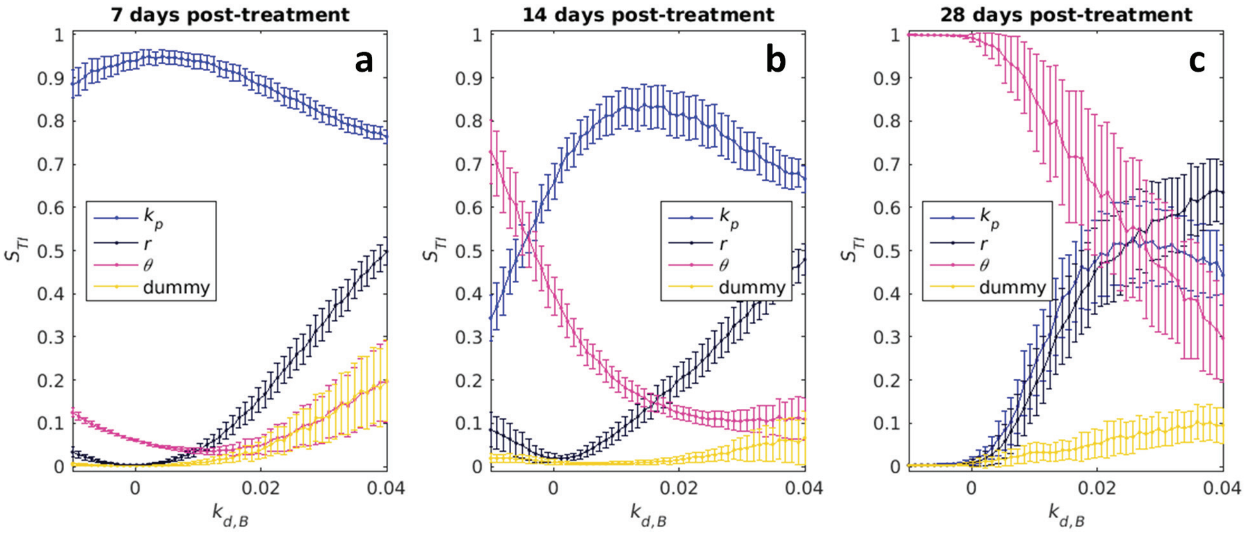

Figure S7: θ fits in a panel of TNBC cell lines as a function of $C_{B,max}$. θ's under Eqs. (5A) – (5B) are fit to each treatment condition as described in Section 2.5 and plotted with 95% confidence intervals against the cell-line specific simulated $C_{B,max}$ from Eqs. (1) – (3). The blue X's, red O's, and green Δ's represent the parameter fits extracted from the 6, 12, and 24 hour exposure time datasets respectively. Model parameters estimated from each exposure time appear to collapse on each other, when described by $C_{B,max}$ – a summary statistic of each treatment condition. Notably, the carrying capacity appears to vary slightly as a function of treatment condition. Additionally, large error bars are observed for high values of $C_{B,max}$. Under these treatment conditions, the cell population does not approach carrying capacity, and the parameter is unable to be resolved.

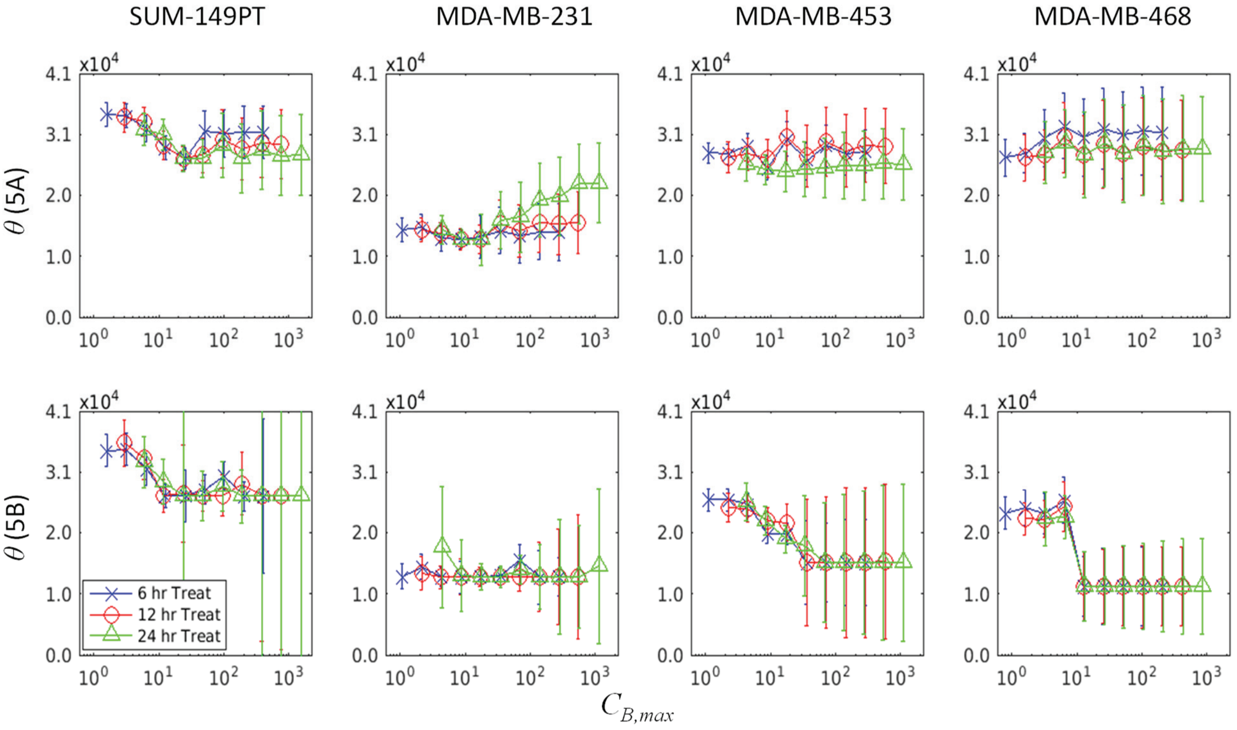

Figure S8: Model weights vary across dose range. Each model (i.e., Eq. (5A) and (5B)) was fit to each treatment condition in the training set. The AIC was calculated for each model fit, and the models were weighted as described in Section 2.5. The blue X's, red O's, and green Δ's represent the parameter fits extracted from the 6-, 12-, and 24-hour exposure time datasets respectively.

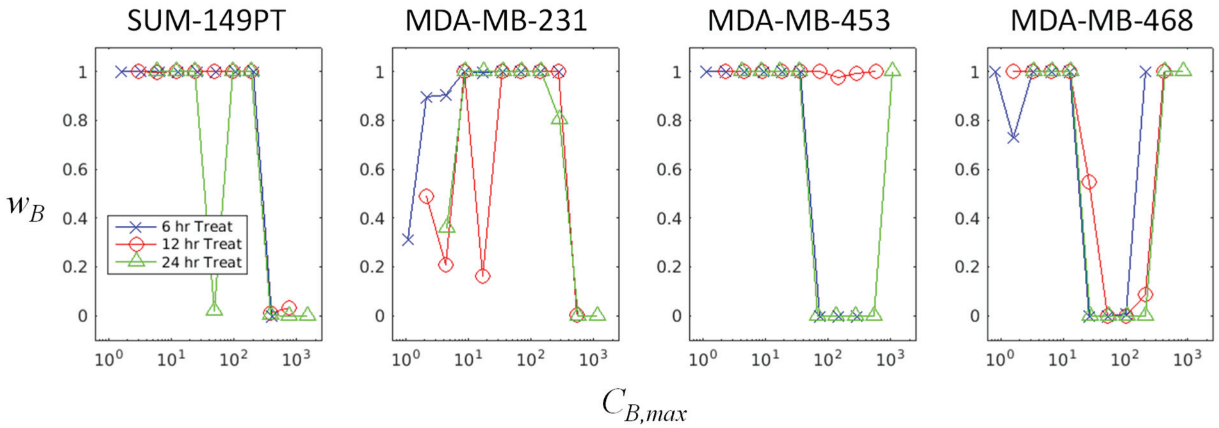

Figure S9: Prediction approach. Model parameters ($k_{d,A}$, θ in Eq. (5A) and $k_{d,B}$, r, θ in Eq. (5B)) were fit to each treatment condition in the training set (a, red X's). These parameter fits were then described by a local regression model (a, black line) to generate parameter estimates for treatments in the test set (a, blue O's). Model weights from the training set (b, red x's) were described by a logistic model (b, black line) to generated weights for the test set (b, blue circles). Final predictions represent a weighted average of each model, and a bootstrap analysis was used to generate a 95% confidence interval for these predictions (red overlay in c).

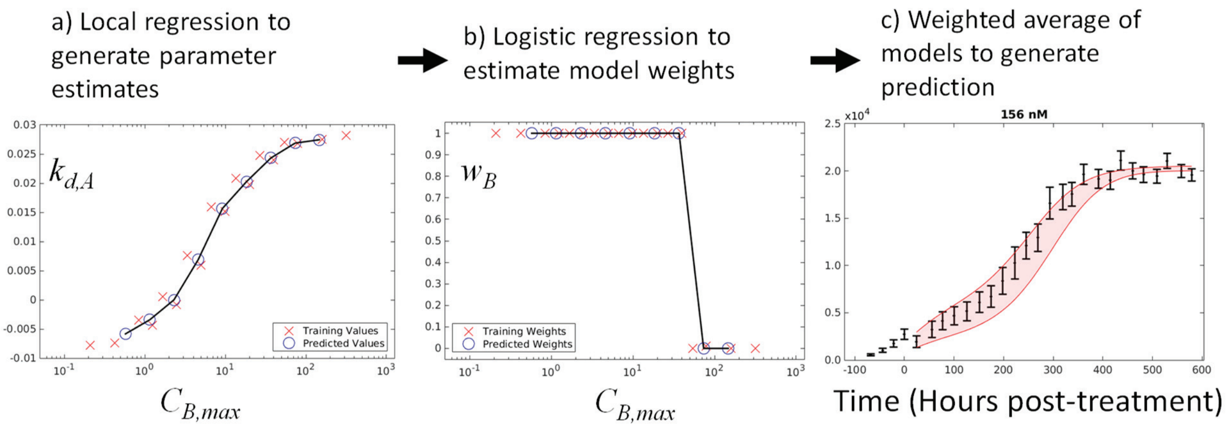

Table S1: Table of model statistics for model fits in SUM-149PT, MDA-MB-231, MDA-MB-453, and MDA-MB-468 cell lines following 12 hour doxorubicin treatment.

	Average % Error of Best-Fit Model (12 hour dataset)			
Concentration (nM)	SUM-149PT	MDA-MB-231	MDA-MB-453	MDA-MB-468
10	7.1	10.3	7.7	7.6
20	7.2	8.3	10.2	6.9
39	5.9	12.1	9.0	11.1
78	5.5	19.1	15.0	21.6
156	10.3	19.4	20.2	35.9
312	19.0	33.1	22.6	32.4
625	31.4	36.9	26.2	34.2
1250	46.6	41.8	30.0	30.2
2500	47.6	32.3	48.9	30.1

Table S2: Table of model statistics for SUM-149PT cell line following 12 and 24 hours of treatment with doxorubicin.

	SUM-149PT							
	12 hour Doxorubicin Treatment				24 hour Doxorubicin Treatment			
	Average % Error		Mean Difference EoE		Average % Error		Mean Difference EoE	
Concentration (nM)	Best-fit	Predicted	Best-fit	Predicted	Best-fit	Predicted	Best-fit	Predicted
10	7.1	7.6	5.8	5.5	6.4	8.1	5.7	6.2
20	7.2	8.1	5.5	5.7	5.6	12.4	4.3	9.3
39	5.9	7.0	3.7	4.2	5.5	12.6	4.9	12.1
78	5.5	7.1	4.6	6.1	10.3	13.3	8.7	13.7
156	10.3	13.0	9.0	15.1	22.7	23.7	12.0	12.6
312	19.0	21.0	21.1	21.7	31.4	35.7	25.2	37.4
625	31.4	33.6	23.0	33.3	50.8	42.7	120.3	89.5
1250	46.6	40.8	125.0	78.8	30.2	34.3	23.1	66.8
2500	47.6	47.7	82.2	75.3	40.0	49.6	32.0	74.6
Average Errors	20.1	20.7	31.1	27.3	22.5	25.8	26.2	35.8

Table S3: Table of model statistics for SUM-149PT cell line following 6 and 12 hours of treatment with doxorubicin.

	SUM-149PT							
	6 hour Doxorubicin Treatment				12 hour Doxorubicin Treatment			
	Average % Error		Mean Difference EoE		Average % Error		Mean Difference EoE	
Concentration (nM)	Best-fit	Predicted	Best-fit	Predicted	Best-fit	Predicted	Best-fit	Predicted
10	5.3	7.6	3.8	7.1	7.1	9.9	5.8	8.9
20	5.3	7.7	3.0	7.2	7.2	7.3	5.5	5.3
39	6.1	7.2	4.6	5.9	5.9	9.8	3.7	8.7
78	6.1	13.7	5.0	5.5	5.5	11.5	4.6	6.3
156	4.6	13.9	4.5	10.3	10.3	10.9	9.0	8.6
312	9.4	15.9	6.8	19.0	19.0	19.2	21.1	22.7
625	15.0	18.8	13.1	31.4	31.4	33.1	23.0	22.1
1250	34.0	41.7	34.3	46.6	46.6	49.0	125.0	112.8
2500	24.4	46.4	61.1	47.6	47.6	50.7	82.2	62.1
Average Errors	12.2	19.2	15.1	20.1	20.1	22.4	31.1	28.6
